# Disturbing Cholesterol/Sphingolipid Metabolism by Squalene Epoxidase Arises Crizotinib Hepatotoxicity

**DOI:** 10.1002/advs.202414923

**Published:** 2025-01-21

**Authors:** Hao Yan, Xiangliang Huang, Yourong Zhou, Yuan Mu, Shaoyin Zhang, Yashi Cao, Wentong Wu, Zhifei Xu, Xueqin Chen, Xiaochen Zhang, Xiaohong Wang, Xiaochun Yang, Bo Yang, Qiaojun He, Peihua Luo

**Affiliations:** ^1^ Center for Drug Safety Evaluation and Research of Zhejiang University College of Pharmaceutical Sciences Zhejiang University Hangzhou 310058 China; ^2^ Department of Thoracic Oncology Hangzhou Cancer Hospital Affiliated Hangzhou First People's Hospital School of Medicine Westlake University Hangzhou 310006 China; ^3^ Department of Medical Oncology The First Affiliated Hospital Zhejiang University School of Medicine Hangzhou 310003 China; ^4^ Zhejiang Cancer Hospital Hangzhou Institute of Medicine (HIM) Chinese Academy of Sciences Hangzhou 310022 China; ^5^ School of Medicine Hangzhou City University Hangzhou 310015 China

**Keywords:** autophagy, crizotinib, hepatotoxicity, lysosomal dysfunction, sphingolipid, squalene epoxidase

## Abstract

Metabolic disorders have been identified as one of the causes of drug‐induced liver injury; however, the direct regulatory mechanism regarding this disorder has not yet been clarified. In this study, a single regulatory mechanism of small molecule kinase inhibitors, with crizotinib as the representative drug is elucidated. First, it is discovered that crizotinib induced aberrant lipid metabolism and apoptosis in the liver. A mechanistic study revealed that crizotinib treatment promoted the accumulation of squalene epoxidase (SQLE) by inhibiting autophagosome‐lysosome fusion which blocked the autophagic degradation of SQLE. A maladaptive increase in SQLE led to disturbances in cholesterol and sphingolipid metabolism via an enzymatic activity‐dependent manner. Abnormal cholesterol results in both steatosis and inflammatory infiltration, and disturbances in sphingolipid metabolism promote cell apoptosis by inducing lysosomal membrane permeabilization. The restoration of the level or activity of SQLE ameliorated steatosis and hepatocyte injury. The autophagy activator known as metformin or the SQLE enzymatic inhibitor known as terbinafine has potential clinical use for alleviating crizotinib hepatotoxicity.

## Graphical abstract

Crizotinib induces excessive accumulation of squalene epoxidase (SQLE) by blocking its autophagic degradation. This accumulation disrupts cholesterol and sphingolipid metabolism, leading to hepatic steatosis or hepatocyte apoptosis, respectively. Autophagy activators, such as metformin, or SQLE enzymatic inhibitors, like terbinafine, possesses the potential clinical use for alleviating crizotinib hepatotoxicity by restoring the level or activity of SQLE.

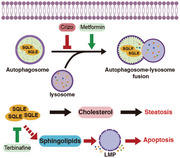

## 1 Introduction

Patients with cancer can experience benefits from treatment with small‐molecule kinase inhibitors (SMKIs); nevertheless, extensive and serious hepatotoxicity has limited their clinical use due to the poor understanding of their toxic mechanism^[^ ^1^ ^]^ and a subsequent lack of intervention strategies. The liver is considered the major metabolic organ, which makes it especially susceptible to damage by drugs; conversely, it can easily cause systemic global metabolism anomalies when liver compliance occurs due to the effects of exogenous drugs. Numerous clinical medications have been shown to induce hepatotoxicity by disturbing metabolic homeostasis, such as via alterations in lipid metabolism, bile acid metabolism, nucleoside metabolism etc.^[^ ^2^ ^]^ Herein, we found that crizotinib‐induced hepatotoxicity was elicited by metabolic abnormalities.

Crizotinib, a multiple‐tyrosine kinase inhibitor of anaplastic lymphoma kinase (ALK), MET, and c‐ros oncogene 1 (ROS1),^[^ ^3^ ^]^ serves as the first‐line treatment for advanced ALK‐positive non‐small‐cell lung cancer (NSCLC).^[^ ^4^ ^]^ However, according to a clinical trial, 55.1% (70/127) of patients experience hepatotoxicity, and the incidence of grade III or grade IV liver injury is 5.5% (7/127), which represents a serious liver complication.^[^ ^5^ ^]^ Crizotinib can cause acute hepatitis and even fatal fulminant hepatic failure. A liver biopsy from one fulminant hepatitis patient revealed predominant inflammatory infiltration, prominent cholestasis, hepatocyte loss and parenchymal collapse.^[^ ^6^ ^]^ Another study has reported that patients can develop hepatic steatosis, which is evident on abdominal imaging.^[^ ^7^ ^]^ All of these clinical findings suggest the complexity and multiple stages of crizotinib hepatotoxicity. To date, the exact mechanism of crizotinib‐induced hepatotoxicity is largely unknown. Based on a previous toxicity study on crizotinib which demonstrated that crizotinib treatment induced massive accumulation of cholesterol in normal cells,^[^ ^8^ ^]^ we questioned whether cholesterol metabolism abnormalities are involved in the hepatotoxicity of this drug.

Cholesterol is an essential component of cellular membranes and is pivotal for maintaining the homeostasis of mammalian cells. However, an excessive amount of cholesterol is generally believed to be positively related to metabolic liver injury, and the critical mechanism of this type of injury is attributed to the extreme damage of functional membrane proteins by cholesterol, which increases membrane rigidity and the induction of mitochondrial stress and these events are followed by reactive oxygen species (ROS) generation and apoptosis activation.^[^ ^9^ ^]^ Moreover, excessive cholesterol in liver can cause steatosis and inflammatory response.^[^ ^10^ ^]^ Cholesterol metabolism is precisely regulated, and this regulation involves the biosynthesis, transport, absorption and conversion of cholesterol into bile acids or steroid hormones.^[^ ^9^ ^]^ The liver plays an important role in cholesterol homeostasis, as it accounts for >50% of systemic cholesterol biosynthesis.^[^ ^11^ ^]^ Some chemicals are reported to induce hepatotoxicity by influencing liver cholesterol biosynthesis, thereby disturbing cholesterol homeostasis.^[^ ^12^ ^]^ Based on this observation, we hypothesized that crizotinib may impact the hepatic cholesterol biosynthesis pathway, thereby leading to cholesterol accumulation and subsequent liver injury.

Cholesterol can be synthesized by hepatocytes and squalene monooxygenase (SQLE) serves as a second rate‐limiting enzyme in cholesterol biosynthesis.^[^ ^13^ ^]^ The primary biological function of SQLE is to catalyze the conversion of squalene to 2,3‐oxidosqualene,^[^ ^14^ ^]^ with the enzyme demonstrating differential roles in cell survival. Specifically, in abnormally proliferating cancer cells, SQLE functions as a prosurvival factor by inhibiting apoptosis and reprogramming metabolism.^[^ ^15^ ^]^ Conversely, in normal cells, a high level of SQLE drives cell fate toward death via the activation of apoptosis and the inhibition of proliferation,^[^ ^16^ ^]^ which was also observed in our study. However, the specific mechanism by which SQLE induces cell apoptosis is still ambiguous. When considering that the accumulation of cholesterol is reported to induce cell apoptosis, whether SQLE induces apoptosis via the activation of cholesterol biosynthesis or through other unknown routes warrants further investigation.

In this study, we revealed that crizotinib can induce aberrant lipid metabolism, autophagy inhibition and apoptosis in hepatocytes. Mechanistically, crizotinib disrupts the homeostasis of cholesterol metabolism in hepatocytes by inhibiting the autophagic degradation of SQLE. An excess amount of SQLE overly increases cholesterol, thus resulting in steatosis and inflammatory infiltration; moreover, sphingosine results in both lysosomal rupture and apoptosis. Notably, the promotion of the fusion of autophagosomes and lysosomes with metformin or the suppression of the enzymatic activity of SQLE with terbinafine significantly alleviates crizotinib‐induced liver injury. Overall, our study characterizes a novel biological function of SQLE in determining cell fate by influencing sphingolipid metabolism and provides new strategies for clinical intervention.

## 2 Results

### 2.1 Crizotinib Induces Aberrant Metabolism, Apoptosis and Autophagy

**Figure 1:**
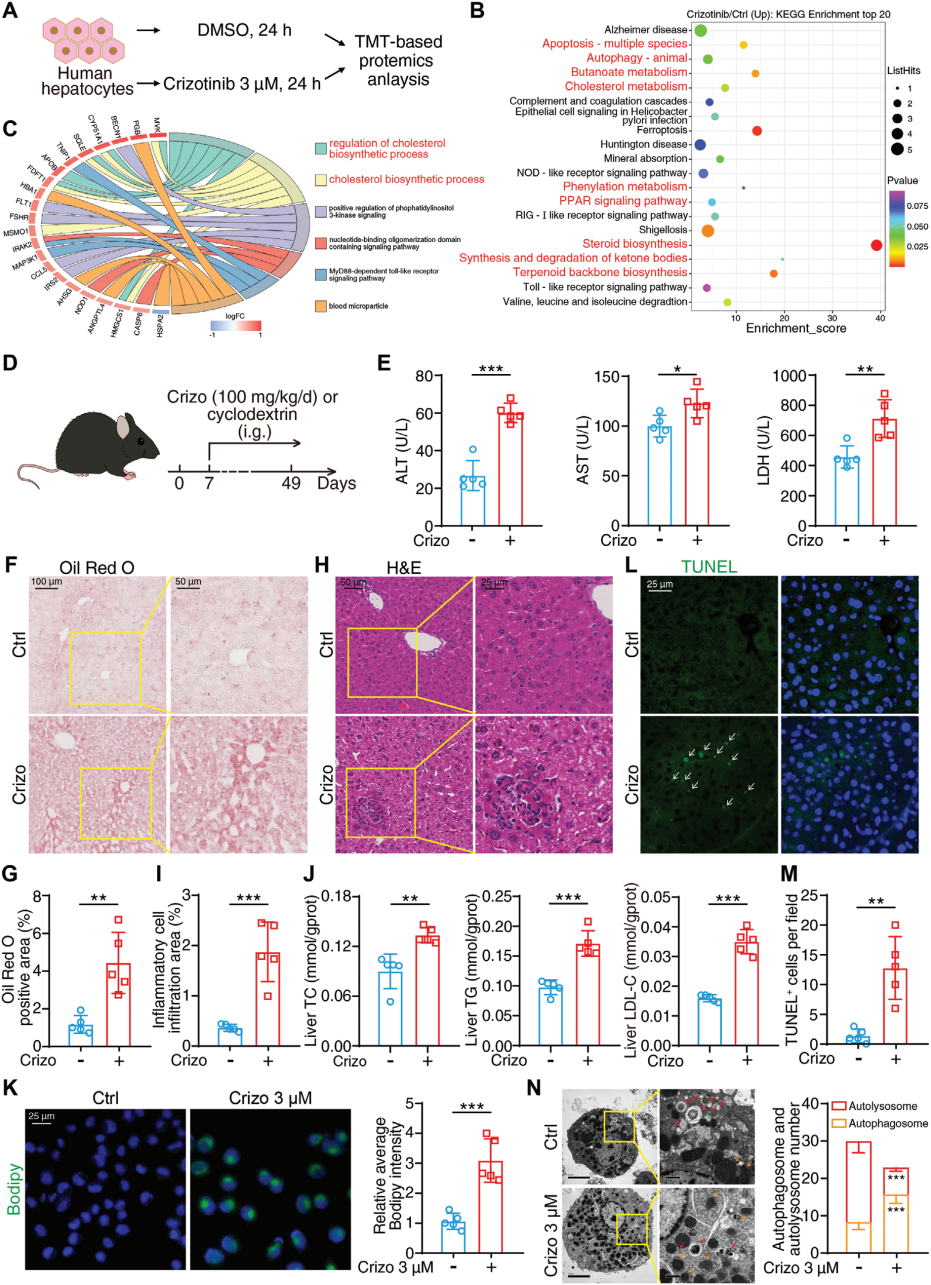
Crizotinib induces aberrant metabolism, apoptosis and autophagy. A–C) TMT quantitative proteomics analysis was used to determine the alteration of whole protein expression after crizotinib treatment. A) The schematic diagram of TMT analysis: HL‐7702 cells were treated with or without 3 µM crizotinib for 24 h followed by TMT labeling and LC‐MS/MS. B) KEGG enrichment analysis of alterable protein expression after crizotinib treatment. The red font highlighted parts mainly divided into metabolism, apoptosis and autophagy pathways. C) GO chord chart analysis of alterable protein expression when exposure to crizotinib. The red font highlighted parts belong to cholesterol metabolism pathway. D–L) In vivo model of crizotinib hepatotoxicity (n = 5 per group). D) The schematic diagram about the construction of in vivo model: 1 week period was provided to allow the animals to adapt to the laboratory environment and then C57BL/6J mice were administered 20% cyclodextrin or crizotinib (100 mg kg^−1^) by gavage daily for 6 weeks. Livers and serum were harvested (n = 5 per group). E) Serum ALT, AST and LDH levels were analyzed. F) Representative images of liver tissues stained with Oil Red O. Scale bar = 100 or 50 µm. G) The Oil Red O‐positive area was analyzed with Fiji software. H) Representative images of liver tissues stained with H&E. Scale bar = 50 or 25 µm. I) The inflammatory cell infiltration area was analyzed with Fiji software. J) Liver TC, TG, and LDL‐C levels were analyzed. K) HL‐7702 cells were treated with or without 3 µM crizotinib for 24 h. Fluorescence microscope images of cells stained with lipid droplet probe (BODIPY) and Hoechst. Scale bar = 25 µm. The average green fluorescence intensity per cell was analyzed and normalized to the control group by Fiji software (n = 5 fields). L) Representative fluorescence microscope images of liver tissues stained with TUNEL and DAPI. Scale bar = 25 µm. White arrows indicated apoptotic cells. M) Quantitative analysis was performed to detect apoptotic cells. N) Mouse primary hepatocytes were treated with or without 3 µM crizotinib for 24 h. Representative images of cellular ultrastructure were detected by transmission electron microscopy. Red arrows denoted autolysosome and orange arrows indicated autophagosome. Quantitative analysis of autophagosomes and autolysosomes per cell was performed (n = 5 cells). The results are presented as the mean ± SD. The *P* value was calculated by Student's *t* test (unpaired, two‐tailed, 2 groups). *P < 0.05; **P < 0.01; ***P < 0.001.

Clinical trial has demonstrated the occurrence of extensive and severe hepatotoxicity in crizotinib‐treated patients.^[^ ^5^ ^]^ To clarify the molecular mechanism of crizotinib hepatotoxicity, we conducted a proteomics study by using TMT labeling and LC‐MS/MS on control hepatocytes and crizotinib‐treated human hepatocytes (**Figure** **1**A). In total, 90 proteins were upregulated after crizotinib treatment, whereas 15 proteins were downregulated (proteins with a fold change ≥ 3/2 or ≤ 2/3 were filtered). Kyoto Encyclopedia of Genes and Genomes (KEGG) enrichment analysis revealed that the altered proteins were mainly enriched in multiple metabolic pathways, apoptosis, and autophagy (Figure 1B). The chord chart about Gene Ontology (GO) analysis revealed evident changes in cholesterol metabolism‐related processes, which mainly concentrated on the cholesterol biosynthetic process (Figure 1C). To better verify the proteomics results, we reproduced the hepatotoxicity of crizotinib in both in vitro and in vivo models. C57BL/6J mice were treated with 20% cyclodextrin or crizotinib (100 mg kg^−1^) by gavage daily for 6 weeks (Figure 1D). The dosage of crizotinib was translated from humans to mice based on the body surface area normalization method and was equivalent to the clinically recommended dose.^[^ ^18^ ^]^ Compared with the control treatment, crizotinib resulted in increased liver injury‐related serum biomarkers, such as alanine aminotransferase (ALT), aspartate aminotransferase (AST), and lactate dehydrogenase (LDH) (Figure 1E), which are clinically matched. When considering that aberrant hepatic cholesterol metabolism usually caused steatosis and inflammatory response,^[^ ^10c^ ^]^ we conducted Oil Red O and hematoxylin and eosin (H&E) staining to detect lipid‐related and potentially subsequent changes. We confirmed increases in the Oil Red O‐positive area and inflammatory cell infiltration area after exposure to crizotinib (Figure 1F–I). Similarly, total cholesterol (TC), triglyceride (TG), and low‐density lipoprotein‐cholesterol (LDL‐C) were also upregulated in the liver after crizotinib treatment (Figure 1J). The abovementioned results were also validated in vitro by using the lipid droplet probe BODIPY. We confirmed that crizotinib markedly triggered lipid accumulation in an immortalized human liver cell line HL‐7702 (with the clinically relevant Cmax of crizotinib being ≈0.91 µM^[^ ^19^ ^]^) (Figure 1K). These results collectively support the hypothesis that crizotinib can induce metabolic anomalies.

In addition to the pathological alteration of metabolic pathways, we investigated apoptosis and autophagy in crizotinib‐induced liver injury based on the proteomics results. Apoptosis is recognized as being a physiological form of programmed cell death^[^ ^20^ ^]^ that is closely associated with drug‐induced liver injury.^[^ ^21^ ^]^ The TdT‐mediated dUTP nick end labeling (TUNEL) assay was used to detect apoptosis; consequently, we demonstrated that crizotinib strongly induced apoptosis in the liver, as indicated by the increase in the number of TUNEL^+^ cells (white arrows) (Figure 1L,M). Moreover, we treated human hepatocytes with different concentrations of crizotinib for 24 h. A light micrography revealed the occurrence of cell death after crizotinib treatment, as demonstrated by the decreased cell number (Figure S1A, Supporting Information). Additionally, the western blot results revealed that crizotinib increased the expression of cleaved poly (ADP‐ribose) polymerase (c‐PARP, which is a marker of apoptosis) in a concentration‐ and time‐dependent manner, which was in accordance with the results of Annexin V‐PI staining combined with flow cytometric analysis, as evidenced by the increase in the apoptotic rate (Figure S1B,C, Supporting Information). Macroautophagy/autophagy is an evolutionarily conserved degradation process that plays a critical role in the maintenance of cellular homeostasis.^[^ ^22^ ^]^ To investigate this process, hepatocytes were treated with or without crizotinib for 24 h; subsequently, transmission electron microscopy (TEM) was utilized to detect the cellular ultrastructure. The results demonstrated that crizotinib treatment led to excessive accumulation of autophagosomes which are characterized by a double‐ or multilayer membrane structure with a tendency to encapsulate cytosolic contents (orange arrows); however, crizotinib treatment decreased the number of autolysosomes, which exhibit a monolayer membrane structure containing degraded cytosolic components (red arrows) (Figure 1N). To further visualize autophagic flux, we infected hepatocytes with the Ad‐mCherry‐GFP‐LC3 virus, in which LC3 was tagged with GFP and mCherry luminescent proteins. The green fluorescence is quenched in lysosomes due to the instability of GFP under acidic conditions, and red fluorescence continues to occur, thereby distinguishing autophagosomes from autolysosomes. Compared with the control group, the number of autophagosomes (yellow puncta) apparently increased after crizotinib treatment (Figure S2A, Supporting Information), thus indicating a blockade at the late stage of autophagy. The intracellular level of LC3‐II (a classic autophagosome marker) demonstrates a decreased intracellular level due to its autophagic degradation after the fusion of autophagosomes and lysosomes. The protein level of LC3‐II was obviously increased upon exposure to crizotinib in various liver cell lines (such as HL‐7702, THLE‐2 and AML12) (Figure S2B, Supporting Information). The abovementioned findings suggest the presence of abundant autophagosomes when autophagosome‐lysosome fusion blockage by crizotinib. The top candidate drug for activating autophagy is metformin, as has been previously reported,^[^ ^23^ ^]^ and metformin was able to restore blocked autophagic flux during crizotinib treatment, as shown by the increased number of autolysosomes (red puncta) and decreased number of autophagosomes (orange puncta) (Figure S2A, Supporting Information). One of our previous studies has demonstrated that the autophagosome‐lysosome fusion is under the regulation of phosphorylated AMPK at Ser485/491, which could be activated by metformin.^[^ ^3^ ^]^ Herein, we revealed that crizotinib significantly downregulated the level of p‐AMPK (S48/491) (Figure S2B, Supporting Information), and this downregulation could be reversed when combined with metformin (Figure S3A, Supporting Information), thereby supporting the existence of blockade of autophagosome‐lysosome fusion.

Taken together, these data suggest that crizotinib can cause aberrant effects with respect to cholesterol metabolism, apoptosis and autophagy inhibition.

### 2.2 Crizotinib Induces Excessive Accumulation of SQLE, Which Leads to Aberrant Metabolism and Apoptosis

**Figure 2:**
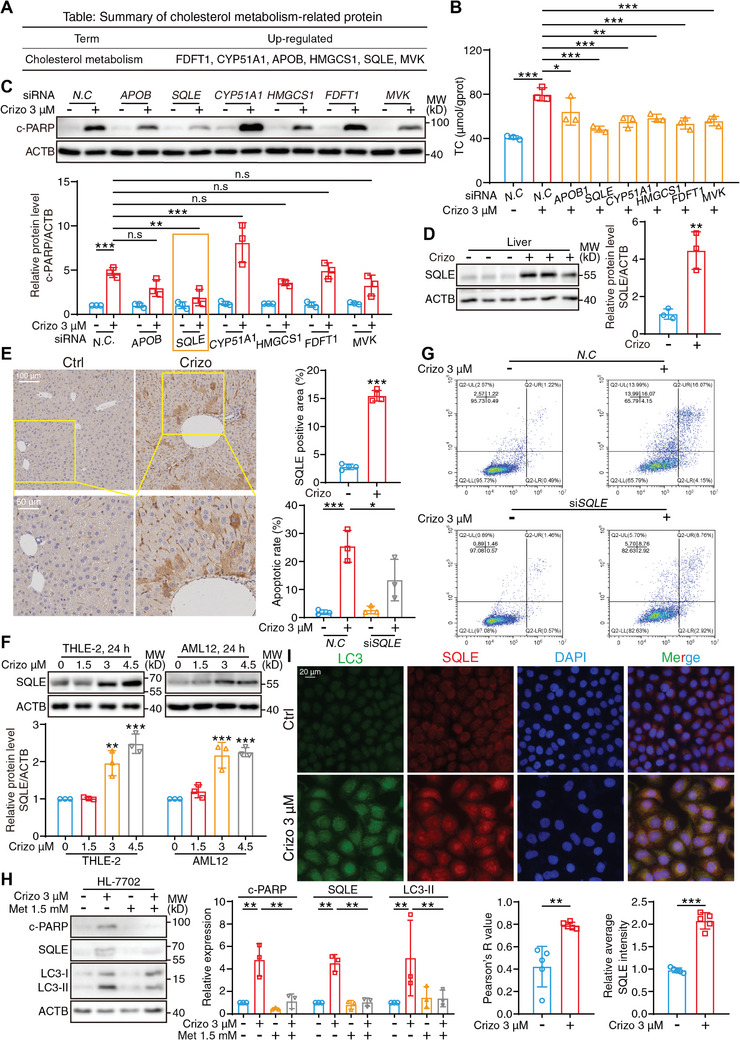
Crizotinib induces the excessive accumulation of SQLE, which leads to aberrant metabolism and apoptosis. A) Summary of upregulated cholesterol metabolism‐related proteins information by TMT‐labeling quantitative proteomics. B,C) HL‐7702 cells were transfected with siRNA against *N.C*, *APOB*, *SQLE*, *CYP51A1*, *HMGCS1*, *FDFT1* and *MVK*, then followed with the treatment of 3 µM crizotinib. B) Total cholesterol within HL‐7702 cells was analyzed (n = 3 independent replicates). C) Relative expression of c‐PARP was analyzed by western blot with ACTB as a loading control (n = 3 independent replicates). D,E) C57BL/6J mice were administered 20% cyclodextrin or crizotinib (100 mg kg^−1^) by gavage daily for 6 weeks. D) Relative expression of SQLE was analyzed by western blot with ACTB as a loading control (n = 3 per group). E) Representative images of immunochemistry with antibody against SQLE from the control group and crizotinib‐treated group, scale bar = 100 µm. The SQLE‐positive area was analyzed with Fiji software (n = 4 per group). F) THLE‐2 cells and AML12 cells were treated with crizotinib in a concentration‐dependent fashion. Relative expression of SQLE was analyzed by western blot with ACTB as a loading control (n = 3 independent replicates). G) HL‐7702 cells were transfected with siRNA against *N.C* or *SQLE* and exposed to 3 µM crizotinib for 24 h. The apoptotic rate was analyzed by flow cytometry combination with Annexin V/PI staining and representative images are shown (n = 3 independent replicates). H) HL‐7702 cells were treated 3 µM crizotinib with or without 1.5 mM metformin for 24 h. Relative expressions of c‐PARP, SQLE, and LC3 were analyzed by western blot with ACTB as a loading control (n = 3 independent replicates). I) HL‐7702 cells were treated with or without 3 µM crizotinib. Representative fluorescent images of cells stained with LC3 (green), SQLE (red) and DAPI (blue) were presented. The Pearson's R value and the relative average red fluorescence intensity per cell normalized to ctrl group were analyzed with Fiji software (n = 5 fields). The results are presented as the mean ± SD. The *P* value was calculated by one‐way ANOVA (Dunnett's multiple comparisons test) for panel (B, C, F, G, and H) or Student's *t* test (unpaired, two‐tailed, 2 groups) for panel (D, E, and I). n.s = no significance; *P < 0.05; **P < 0.01; ***P < 0.001.

With the aim of developing more precise and effective intervention strategies, it is important to identify the central regulatory target involved in crizotinib hepatotoxicity. A review of the TMT‐labeling proteomics results revealed that the steroid biosynthesis pathway exhibited the highest enrichment score with a high degree of confidence (*P* value < 0.025) in the KEGG analysis. Moreover, GO chord chart analysis revealed that the most significantly altered biological process was associated with cholesterol biosynthesis. The increased cholesterol level was confirmed in human primary hepatocytes and mouse primary hepatocytes with crizotinib treatment (Figure S4A,B, Supporting Information). After integrating the KEGG and GO protein enrichment analyses, a total of 6 proteins were filtered for subsequent investigation, including apolipoprotein B (APOB), SQLE, cytochrome P450 family 51 subfamily A member 1 (CYP51A1), 3‐hydroxy‐3‐methylglutaryl‐CoA synthase 1 (HMGCS1), farnesyl‐diphosphate farnesyltransferase 1 (FDFT1) and mevalonate kinase (MVK), which are involved in the differential cholesterol metabolism process (**Figure** **2**A). Small interfering RNA (siRNA) was used to screen the critical factors mediating crizotinib hepatotoxicity. The knockdown efficiency of the siRNAs was tested by quantitative real‐time polymerase chain reaction (qPCR), which demonstrated that all of the siRNAs effectively silenced corresponding target candidates (Figure S5A, Supporting Information). Notably, the suppression of the expression of each candidate gene was able to partially ameliorate crizotinib‐induced cholesterol upregulation, thus remedying metabolic abnormalities, with *SQLE* knockdown resulting in the best intervention effect (Figure 2B). When considering that high cholesterol has been reported to induce apoptosis,^[^ ^24^ ^]^ we assumed that the increased cholesterol biosynthetic process activated apoptosis via intermediary cholesterol. However, the use of western blot analysis for the investigation of c‐PARP expression seems to be ambiguous. The results revealed that the degree of cholesterol decrease after siRNA silencing was mismatched with the change in apoptosis‐related protein, thereby suggesting that cholesterol itself may not be the main contributor to crizotinib‐induced cell apoptosis (Figure 2C). Intriguingly, compared with the other candidates, the downregulation of SQLE resulted in the most prominent apoptosis‐rescue effect when compared with other candidates. Based on the abovementioned analysis, we speculated that SQLE may serve as the central regulator of crizotinib hepatotoxicity. Subsequently, we proceeded to examine the protein level of SQLE in vivo via both western blot and immunochemistry assays and observed that crizotinib obviously upregulated SQLE (Figure 2D,E). In addition, various in vitro cell models demonstrated that crizotinib markedly increased the SQLE protein level (Figure 2F; Figure S6A,B, Supporting Information). Moreover, flow cytometry analysis, followed by Annexin V‐PI staining, further revealed that the silencing of *SQLE* dramatically reversed the increase in the rate of apoptosis caused by crizotinib to a normal level, thereby confirming the relationship between SQLE and apoptosis (Figure 2G). We have also observed a positive correlation between SQLE and c‐PARP in human primary hepatocytes and mouse primary hepatocytes (Figure S7A,B, Supporting Information).

After synthesizing the abovementioned information, we considered the accumulated SQLE to be the central regulator of crizotinib hepatotoxicity. Therefore, we investigated the manner in which crizotinib induced the accumulation of SQLE and aimed to identify a potential strategy based on protein homeostasis regulation. First, we found that crizotinib had little effect on the transcriptional level of SQLE both in vitro and in vivo (Figure S8A,B, Supporting Information), thus excluding the possibility of transcriptional activation. Second, HL‐7702 cells were pretreated with the protein biosynthesis inhibitor cycloheximide, followed by western blot analysis, which revealed that crizotinib substantially increased the half‐life of SQLE (Figure S8C, Supporting Information). Based on this result, we inferred that the increase in SQLE may be attributed to the inhibition of degradation. The intracellular degradation pathway can be divided into the ubiquitin‐proteasome pathway and the autophagy‐lysosome pathway,^[^ ^25^ ^]^ both of which have been previously reported to be involved in the stability regulation of SQLE.^[^ ^26^ ^]^ The 26S proteasome inhibitor MG‐132 was utilized to inhibit proteasomal degradation, and this inhibitor was able to aggravate crizotinib‐induced SQLE upregulation (Figure S8D, Supporting Information). However, the inhibition of intracellular autophagy degradation by using the lysosomal inhibitor bafilomycin A1 did not affect the accumulation of SQLE in the context of crizotinib (Figure S8E, Supporting Information). These results suggest that the inhibition of autophagic degradation was the main contributor to crizotinib‐induced SQLE accumulation. Hence, the autophagy activator metformin was introduced to remedy the blockade of autophagy flux, which was able to downregulate SQLE and ultimately relieve crizotinib‐induced cell apoptosis (Figure 2H). A similar result was obtained with another autophagy activator known as rapamycin (Figure S8F, Supporting Information). In addition, an immunofluorescence assay was conducted to detect the intracellular distribution of SQLE and confirmed that due to crizotinib treatment, the colocalization between SQLE and LC3 was obviously enhanced, as evidenced by the increased Pearson's R value and relative SQLE fluorescence intensity (Figure 2I). These results indicated that SQLE mainly accumulated in autophagosomes when exposed to crizotinib. Notably, we also detected ubiquitin anchoring in SQLE and confirmed that crizotinib promoted the ubiquitin modification of SQLE. The ubiquitin located in SQLE serves as an “eat me” signal and is essential for recognition between the substrate and the autophagy receptor.^[^ ^27^ ^]^ Thus, the increase in the level of ubiquitinated SQLE reflected the considerable aggregation of SQLE in autophagosomes (Figure S8G, Supporting Information), and the enzymatic domain within SQLE is not affected by the ubiquitin‐modification on its stability regulation domain according to the structural analysis on UniProt database. Overall, we concluded that crizotinib blocks autophagosome‐lysosome fusion and subsequently leads to the excessive accumulation of SQLE, which excessively triggers the biosynthesis of cholesterol and cell apoptosis, thus leading to hepatotoxicity.

### 2.3 Downregulation of SQLE Relieves Crizotinib‐Induced Hepatotoxicity

**Figure 3:**
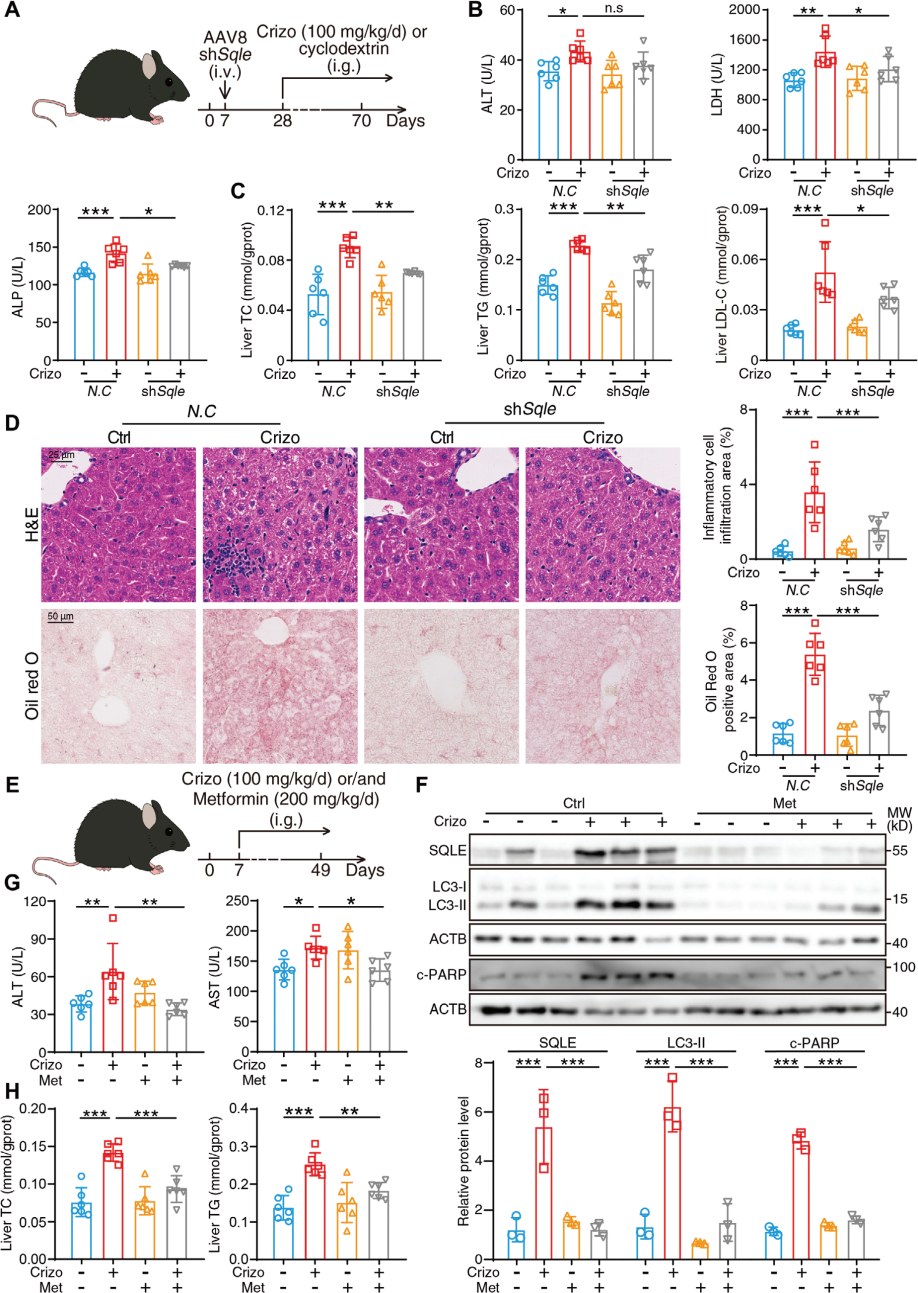
Downregulation of SQLE relieves crizotinib‐induced hepatotoxicity. A–D) In vivo model of *Sqle* knockdown effect on crizotinib treatment (n = 6 per group). A) The schematic diagram about the construction of in vivo model: 1 week period was provided to allow the animals adapt to the laboratory environment and then C57BL/6J mice were injected with AAV8‐TBG promoter‐*N.C* or AAV8‐TBG promoter‐sh*Sqle* via tail vein. After 3 weeks, the mice were administered 20% cyclodextrin or crizotinib (100 mg kg^−1^) by gavage daily for 6 weeks. Livers and serum were harvested (n = 6 per group). B) Serum ALT, LDH, and ALP levels were analyzed. C) Hepatic TC, hepatic TG, and hepatic LDL‐C levels were analyzed. D) Representative images of liver tissues stained with H&E or Oil Red O, scale bar = 25 or 50 µm, respectively. The inflammatory cell infiltration area and Oil Red O‐positive area were analyzed with Fiji software. E–H) In vivo model of metformin intervention with crizotinib‐induced hepatotoxicity (n = 6 per group). E) The schematic diagram about construction of in vivo model: 1 week period was provided to allow the animals to adapt to the laboratory environment and then C57BL/6J mice were administered 20% cyclodextrin, 100 mg kg^−1^ crizotinib, 200 mg kg^−1^ metformin or crizotinib plus metformin by gavage daily for 6 weeks. Livers and serum were harvested. F) Relative expressions of SQLE, LC3, and c‐PARP were analyzed by western blot with ACTB as a loading control. G) Serum ALT and AST levels were analyzed. H) Liver TC and TG levels were analyzed. The results are presented as the mean ± SD. The *P* value was calculated by one‐way ANOVA (Dunnett's multiple comparisons test). n.s = no significance; *P < 0.05; **P < 0.01; ***P < 0.001.

Based on the in vitro results, we sought to investigate the role of SQLE in the hepatotoxicity of crizotinib in vivo. First, we applied AAV8‐TBG promoter‐*Sqle* shRNA (a liver‐specific targeting AAV8 vector with a TBG promoter) to suppress the expression of SQLE in mice, followed by gavage with 20% cyclodextrin or crizotinib (100 mg kg^−1^) for 6 weeks (**Figure** **3**A). We first performed western blotting to analyze the knockdown efficiency of *Sqle*, as the results demonstrated that AAV8‐carried *Sqle* shRNA obviously inhibited the expression of SQLE (Figure S9A, Supporting Information). Notably, we discovered that the suppression of SQLE reversed crizotinib‐induced cell apoptosis, as evidenced by the results of the analysis of c‐PARP expression and TUNEL assays (Figure S9A,B, Supporting Information). Furthermore, the detection of serum biomarkers of liver injury revealed that the elevated ALT, LDH and ALP levels were reversed by the knockdown of *Sqle* (Figure 3B). Moreover, the elevated indicators of abnormal liver metabolism due to treatment with crizotinib (including TC, TG and LDL‐C) could also be downregulated after *Sqle* knockdown (Figure 3C), which is consistent with the transcriptional alteration of lipid synthesis indicators such as *Fasn* and *Srebp1* (Figure S9C, Supporting Information). Moreover, H&E staining and Oil Red O staining revealed that *Sqle* knockdown ameliorated inflammatory cell infiltration and accumulation of lipid droplets in liver tissue caused by crizotinib (Figure 3D).

The in vitro results demonstrated that crizotinib‐induced blockade of autophagosome‐lysosome fusion is the major mechanism of SQLE accumulation, which prompted us to investigate this process in vivo. We subsequently selected metformin as an autophagy activator for the treatment of mice, because of its comprehensive drug safety evaluation, with few adverse events being reported.^[^ ^28^ ^]^ C57BL/6J mice were treated with crizotinib (100 mg kg day^−1^) or/and metformin (200 mg kg day^−1^) for 6 weeks (Figure 3E). Western blot analysis of mouse livers revealed that metformin remarkably relieved crizotinib‐induced autophagosome‐lysosome fusion inhibition, as evidenced by changes in the LC3‐II protein level. Furthermore, metformin reduced the elevated expression levels of SQLE and c‐PARP to normal levels, which supported the hypothesis that the autophagy inhibition provoked by crizotinib was the cause of SQLE accumulation and cell apoptosis (Figure 3F). Additionally, we observed that metformin was able to significantly reverse crizotinib‐induced ALT and AST upregulation as well as liver TC and TG levels (Figure 3G,H), thereby indicating that the restoration of autophagy by metformin may serve as an available strategy for treating crizotinib‐induced hepatic injury and metabolic abnormalities. In conclusion, the downregulation of SQLE by both genetic inhibition and pharmacological autophagy activation can remedy crizotinib‐induced hepatotoxicity.

### 2.4 SQLE is Associated with Extensive Metabolite Changes

**Figure 4:**
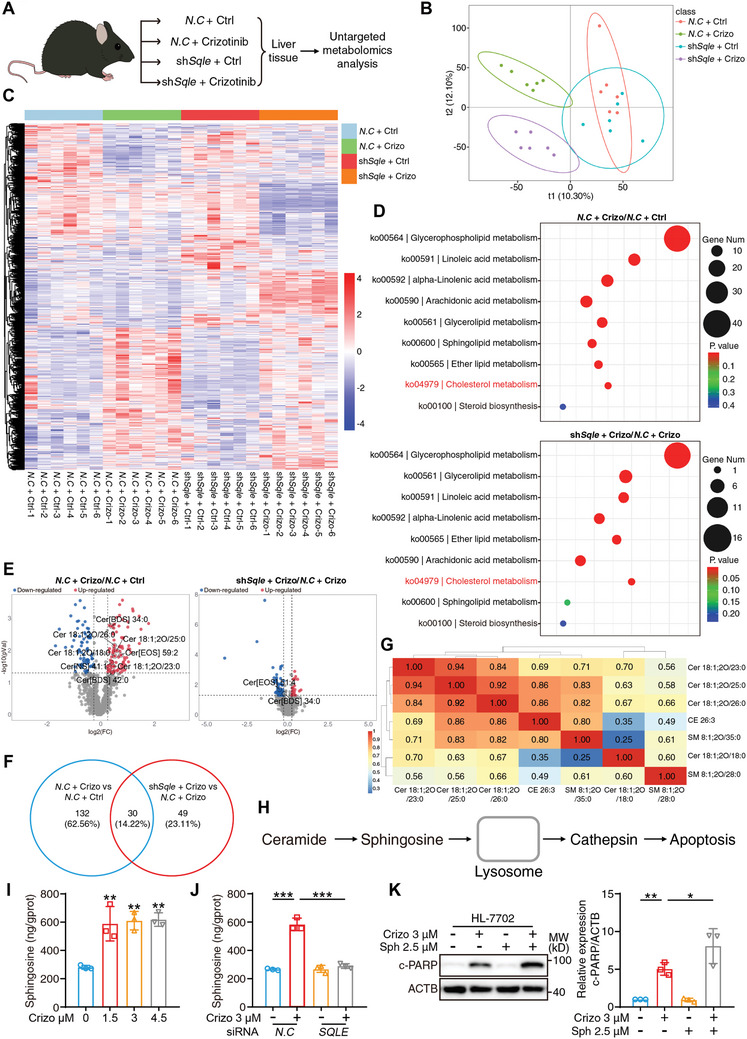
SQLE is associated with extensive metabolite changes. A–G) Untargeted metabolomic analysis was used to determine the alteration of hepatic metabolites after crizotinib treatment as well as after *Sqle* knockdown when simultaneously provided with crizotinib treatment (n = 6 per group). The differential metabolites were identified according to the following rule: 1) the ratio of fold change ≥ 2 or ≤ 1/2; 2) *P* value < 0.05; 3) (Variable Important for the Projection) VIP value ≥ 1. A) The schematic diagram of untargeted metabolomic analysis: The *Sqle* knockdown and drug treatment of C57BL/6J mice had been mentioned above and the liver tissues were harvested for the further untargeted metabolic analysis. The mice could be divided into four groups: *N.C* + Ctrl, *N.C* + Crizotinib, sh*Sqle* + Ctrl, and sh*Sqle* + Crizotinib. B) PLS‐DA analysis of metabolites expression within four groups. C) Heatmap analysis of metabolites expression within four groups, colors depicted the intensity of response. D) KEGG analysis of differentially expressed metabolites between *N.C* + Crizotinib and *N.C* + Ctrl as well as sh*Sqle* + Crizotinib and sh*Sqle* + Ctrl. E) Volcano plot analysis visualized the differentially expressed metabolites between *N.C* + Crizotinib and *N.C* + Ctrl as well as sh*Sqle* + Crizotinib and sh*Sqle* + Ctrl. F) Venn diagram of differentially expressed metabolites by the indicated pairwise comparison (*N.C* + Crizotinib/*N.C* + Ctrl and sh*Sqle* + Crizotinib/sh*Sqle* + Ctrl). G) The correlation analysis of metabolites enriched into cholesterol metabolism and sphingolipid metabolism pathway; colors represented the intensity of response. H) The schematic diagram regarding how sphingolipid belongings resulted in apoptosis: ceramide was converted to sphingosine which preferentially concentrated in lysosomes and led to the release of cathepsin finally inducing apoptosis. I) HL‐7702 cells were treated with crizotinib in a concentration‐dependent manner. The intracellular levels of sphingosine were analyzed by ELISA assay (n = 3 independent replicates). J) HL‐7702 cells were transfected with siRNA against *N.C* or *SQLE* and exposed to 3 µM crizotinib for 24 h. The intracellular levels of sphingosine were analyzed by ELISA assay (n = 3 independent replicates). K) HL‐7702 cells were treated with 3 µM crizotinib with or without 2.5 µM sphingosine for 24 h. The expression of c‐PARP was analyzed with western blot with ACTB as a loading control (n = 3 independent replicates). The results are presented as the mean ± SD. The *P* value was calculated by one‐way ANOVA (Dunnett's multiple comparisons test). *P < 0.05; **P < 0.01; ***P < 0.001.

The abovementioned results suggest that SQLE is the central regulator of crizotinib hepatotoxicity, which is achieved by disrupting cholesterol homeostasis and inducing cell apoptosis. However, the mechanism by which SQLE affects cell apoptosis in the liver is still largely unknown. Given that SQLE plays a foundational role in metabolism, we proceeded to assess whether SQLE induced alterations in metabolites, thereby activating cell apoptosis. Untargeted metabolomic analysis was conducted to compare the metabolites among the following four groups: *N.C* plus Ctrl, *N.C* plus Crizotinib, sh*Sqle* plus Ctrl and sh*Sqle* plus Crizotinib (**Figure** **4**A). Partial least squares discriminant analysis (PLS‐DA) revealed clear group separations between the *N.C* plus Ctrl group and the *N.C* plus Crizotinib group, as well as between the sh*Sqle* plus Ctrl group and the sh*Sqle* plus Crizotinib group, thus indicating that crizotinib induced extensive and significant changes in metabolites that could be reversed by the knockdown of *Sqle* (Figure 4B). Clustering analysis using heatmap visualization of averaged group intensities revealed alterations in metabolites after different treatments which were mainly clustered into fatty acyls, glycerolipids, glycerophospholipids, prenol lipids, sphingolipids, and sterol lipids (Figure 4C; Figure S10A, Supporting Information). KEGG analysis comparing the *N.C* plus Ctrl group with the *N.C* plus Crizotinib group revealed that crizotinib treatment significantly increased the enrichment of the steroid biosynthesis and cholesterol metabolism pathways (*P* value < 0.01). Intriguingly, when the sh*Sqle* plus Crizotinib group was compared with the sh*Sqle* plus Ctrl group, KEGG analysis also confirmed the enrichment of steroid metabolism, in which cholesterol metabolites were obviously downregulated after the knockdown of *Sqle* (Figure 4D), thus confirming that crizotinib induced the alteration of intrinsic metabolites associated with SQLE. Furthermore, we observed that crizotinib treatment alone led to variations in steatosis‐related metabolic pathways, including glycerophospholipid metabolism and linoleic acid metabolism etc., which could be partially reversed by *Sqle* knockdown (Figure 4D). This change in steatosis‐related metabolites may be due to fluctuations in the intracellular cholesterol content. A volcano plot was generated to visualize the relative changes in specific metabolites, and a Venn diagram was also generated to visualize the joint alteration in metabolites when the *N.C* plus Ctrl group was compared with the *N.C* plus Crizotinib group, as well as when the sh*Sqle* plus Crizotinib group was compared with the sh*Sqle* plus Ctrl group (Figure 4E,F). By integrating the two statistical strategies, we concluded that a total of 30 metabolites significantly varied after *Sqle* knockdown under the treatment of crizotinib. Collectively, the abovementioned results support the view that crizotinib induces extensive and global changes in metabolic profile and that SQLE serves as the central regulator of crizotinib‐induced aberrant metabolism.

Sphingolipids have been previously reported to mediate cell apoptosis, and the intracellular ratio of proapoptotic sphingosine and ceramide to antiapoptotic sphingosine‐1‐phosphate determines cell fate.^[^ ^29^ ^]^ Notably, crizotinib obviously changed the sphingolipid metabolism pathway, which was also enriched after *Sqle* knockdown (Figure 4D). Specifically, various ceramide subtypes were measurably upregulated by treatment with crizotinib, which was reversed by *Sqle* knockdown (Figure 4E). A correlation heatmap revealed a positive relationship between cholesterol metabolites and ceramide metabolites, thereby indicating that the induction of apoptosis by SQLE may be attributed to increased ceramide levels (Figure 4G). The analysis of metabolic processes revealed the conversion of ceramide to sphingosine, which is preferentially enriched in lysosomes, thus leading to the leakage of proteolytic enzymes and subsequent apoptosis (Figure 4H). Subsequently, we validated the potential mechanism of SQLE‐induced cell apoptosis in vitro. First, an ELISA was conducted to detect the intracellular sphingosine level, which confirmed that crizotinib dramatically promoted the upregulation of sphingosine in immortalized cell line and primary hepatocytes (Figure 4I; Figure S11A,B, Supporting Information). Afterwards, by using siRNA to achieve the knockdown of *SQLE*, we demonstrated that the increase in sphingosine was caused by the excess SQLE (Figure 4J). In addition, exogenous supplementation with sphingosine aggravated crizotinib‐induced apoptosis (Figure 4K). In view of that lysosome was the major metabolism site where sphingomyelin and glycosphingolipid were catabolized into ceramide and further into sphingosine, the sphingosine was then shuttled from the lysosome to the endoplasmic reticulum through cholesterol or other sterol transporter proteins and metabolized into sphingosine‐1‐phosphate.^[^ ^30^ ^]^ Of note, it was intriguing to note that SQLE was transported toward lysosome after crizotinib treatment, as evidenced by the significant increment of colocalization between SQLE and LAMP1 (the lysosome‐associated membrane glycoprotein 1) (Figure S12A, Supporting Information). This observation suggested that crizotinib‐induced SQLE accumulation through autophagosome‐lysosome fusion blockage could provide spatial connection between SQLE and lysosome. Although the SQLE protein could not finally enter the lysosome, the excessive cholesterol was able to diffuse from the autophagosome to lysosome and competitively inhibit the sphingosine shuttle protein, thus resulting in the lysosomal accumulation of ceramide and sphingosine, which was also proved by the decreased level of their metabolite, sphingosine‐1‐phosphate, after crizotinib treatment (Figure S10A, Supporting Information). Taken together, these results provide evidence that excessive accumulation of SQLE may lead to extensive fluctuation of metabolites, and it is noteworthy that locally accumulation of cholesterol can influence the metabolism of ceramide and sphingosine, which transmits death signals to downstream apoptosis effectors.

### 2.5 Considerable Accumulation of Damaged Lysosomes Induces Apoptosis

**Figure 5:**
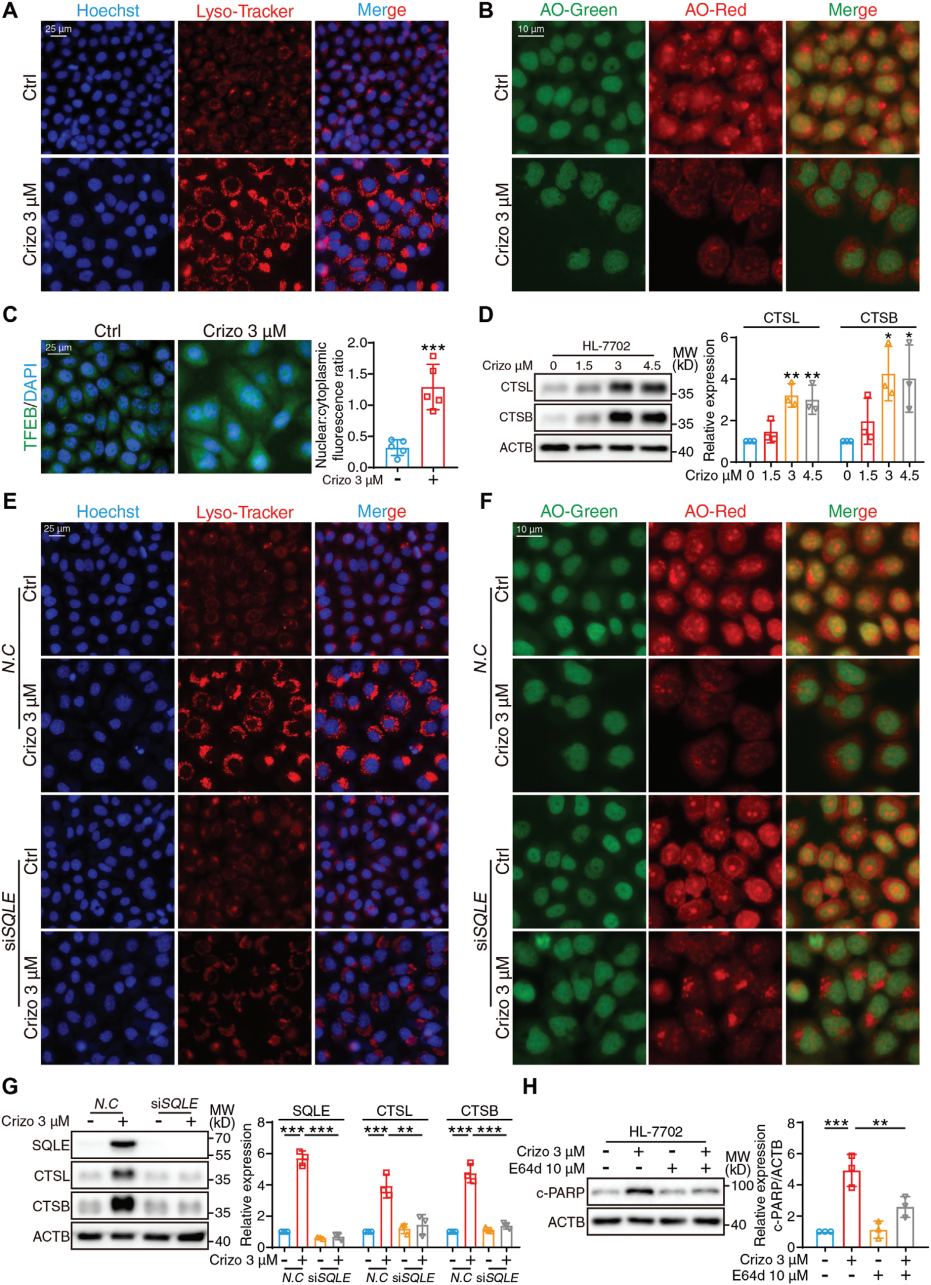
Considerable accumulation of damaged lysosomes induces apoptosis. A–C) HL‐7702 cells were treated with or without 3 µM crizotinib for 24 h. A) Representative fluorescent images of cells stained with Hoechst (blue) and lysosome‐tracker probes (Red) were presented. Scale bar = 25 µm. B) Representative fluorescent images of cells stained with AO were shown by 488 and 594 nm fluorescent channels. Scale bar = 10 µm. C) Representative fluorescent image of cells stained DAPI (blue) and TFEB (green) were presented. Scale bar = 25 µm. The nuclear/cytoplasmic fluorescent ratio of TFEB was analyzed with Fiji software (n = 5 fields). D) HL‐7702 cells were treated with crizotinib in a concentration‐dependent manner. Relative expressions of CTSL and CTSB were analyzed by western blot with ACTB as a loading control (n = 3 independent replicates). E–G) HL‐7702 cells were transfected with siRNA against *N.C* or *SQLE* and exposed to 3 µM crizotinib for 24 h. E) Representative fluorescent images of cells stained with Hoechst (blue) and lysosome‐tracker probes (Red) were presented. Scale bar = 25 µm. F) Representative fluorescent images of cells stained with AO were shown by 488 and 594 nm fluorescent channels. Scale bar = 10 µm. G) Relative expressions of SQLE, CTSL, and CTSB were analyzed by western blot with ACTB as a loading control (n = 3 independent replicates). H) HL‐7702 cells were treated with 3 µM crizotinib with or without 10 µM E64d for 24 h. Relative expression of c‐PARP was analyzed by western blot with ACTB as a loading control (n = 3 independent replicates). The results are presented as the mean ± SD. The *P* value was calculated by one‐way ANOVA (Dunnett's multiple comparisons test) for panel (D, G, and H) or Student's *t* test (unpaired, two‐tailed, 2 groups) for panel (C). *P < 0.05; **P < 0.01; ***P < 0.001.

Thus far, we have shown that the upregulation of ceramide and its metabolite sphingosine is associated with crizotinib‐induced cell apoptosis, which prompted us to explore the manner in which sphingolipids activate apoptosis. When considering blockade of autophagosome‐lysosome fusion in response to crizotinib, we speculated that excessive accumulation of damaged organelles occurred due to impaired clearance. Moreover, sphingosine was shown to destroy the lysosomal membrane and lead to lysosome damage. Therefore, we first introduced a lysosome‐tracker to mark intracellular lysosomes and found that crizotinib treatment significantly increased the number of lysosomes, as visualized by the considerable increase in the number of red puncta (**Figure** **5**A). The acridine orange (AO) uptake method was subsequently applied to monitor lysosomal integrity, and the results revealed a decrease in red fluorescence intensity, thus indicating lysosomal membrane rupture, which resulted in the release of lysosome‐loaded AOs (Figure 5B). Furthermore, the nuclear distribution of transcription factor EB (TFEB) is considered a feedback event when lysosomal damage occurs,^[^ ^31^ ^]^ and an immunofluorescence assay revealed the significant translocation of TFEB from the cytoplasm to the nucleus, which also supported the occurrence of lysosomal damage after crizotinib treatment (Figure 5C). Disruption of the lysosomal membrane structure tends to cause leakage of proteolytic enzymes, such as cathepsin L (CTSL) and cathepsin B (CTSB). Western blot analysis demonstrated that crizotinib induced the upregulation of CTSL and CTSB in a dose‐dependent manner (Figure 5D), which was attributed to the considerable accumulation of damaged lysosomes. Notably, the inhibition of SQLE by using siRNA interference reversed the crizotinib‐induced increase in the number of lysosomes and partially restored the integrity of the lysosomes (Figure 5E,F). In addition, the increased expression of CTSL and CTSB was reversed by *SQLE* knockdown (Figure 5G), which was in accordance with the results of combination with the cysteine protease inhibitor E64d (Figure 5H). Based on the abovementioned results, we concluded that crizotinib induces the upregulation of sphingosine by SQLE and that sphingosine causes subsequent lysosome damage. Due to the inhibition of autophagosome‐lysosome fusion, damaged lysosomes cannot be removed in a timely manner, which further aggravates the leakage of CTSL and CTSB, followed by cell apoptosis.

### 2.6 The SQLE Enzymatic Inhibitor Terbinafine Interferes with the Hepatotoxicity of Crizotinib

**Figure 6:**
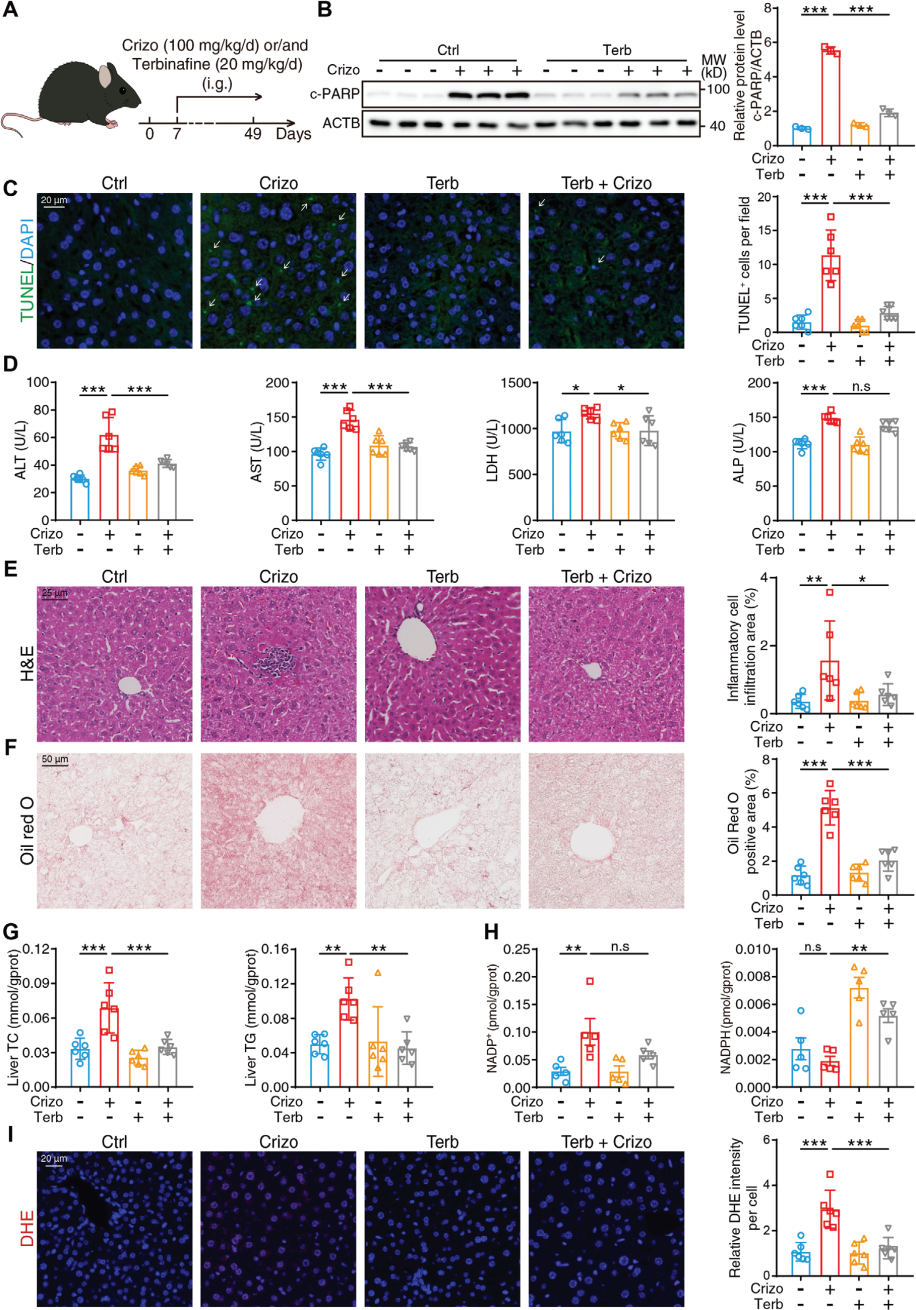
The SQLE enzymatic inhibitor terbinafine interferes with hepatotoxicity of crizotinib. A–F) In vivo model of terbinafine intervention with crizotinib‐induced hepatotoxicity (n = 6 per group). A) The schematic diagram about construction of in vivo model: 1 week period was provided to allow the animals to adapt to the laboratory environment and then C57BL/6J mice were administered 20% cyclodextrin, 100 mg kg^−1^ crizotinib, 20 mg kg^−1^ terbinafine or crizotinib plus terbinafine by gavage daily for 6 weeks. Livers and serum were harvested (n = 6 per group). B) Relative expression of c‐PARP was analyzed by western blot with ACTB as a loading control. C) Representative fluorescent image of liver tissues stained with TUNEL and DAPI. White arrows indicated apoptotic cells. The number of TUNEL^+^ cells per field was calculated. D) Serum ALT, AST, LDH, and ALP levels were analyzed. E) Representative images of liver tissues stained with H&E, scale bar = 25 µm. The inflammatory cell infiltration area was analyzed with Fiji software. F) Representative images of liver tissues stained with Oil Red O, scale bar = 50 µm. The Oil Red O‐positive area was analyzed with Fiji software. G) Liver TC and TG levels were analyzed. H) Hepatic NADP^+^ and NADHP levels were measured. I) Representative fluorescent image of liver tissues stained with DHE and DAPI. The relative DHE florescent intensity was analyzed with Fiji software. The results are presented as the mean ± SD. The *P* value was calculated by one‐way ANOVA (Dunnett's multiple comparisons test). n.s = no significance; *P < 0.05; **P < 0.01; ***P < 0.001.

The abovementioned studies collectively reveal that the accumulation of SQLE induced by crizotinib leads to hepatic metabolism abnormalities and cell apoptosis, which mediates crizotinib hepatotoxicity. When considering the canonical enzyme function of SQLE, which catalyzes the conversion of squalene, we aimed to investigate whether the enzymatic activity of SQLE is crucial for hepatotoxicity induction. According to reported research, Y195 located at SQLE is pivotal for its enzyme activity, and a conservative Y195F mutation results in a > 90% loss of catalytic activity.^[^ ^13^ ^]^ Furthermore, we constructed SQLE activity‐deficient plasmids (SQLE‐Y195F‐Flag), and HL‐7702 cells were concurrently transfected with mutant (Mut) or wild‐type (WT)‐SQLE plasmids. Western blot analysis revealed that transfection with different amounts of WT‐SQLE plasmids directly increased c‐PARP, whereas transfection with SQLE‐Mut plasmids did not affect the expression of c‐PARP (Figure S13A, Supporting Information). Hence, the abovementioned findings suggest potential interventions via the targeting of the enzymatic activity of SQLE. We subsequently introduced the reported noncompetitive SQLE inhibitor terbinafine and combined it with crizotinib both in vitro and in vivo. The in vitro results demonstrated that terbinafine effectively inhibited the upregulation of c‐PARP caused by crizotinib without affecting the protein level of SQLE (Figure S13B, Supporting Information). We then assessed the effect of terbinafine on crizotinib hepatotoxicity in vivo, and C57BL/6J mice were treated with crizotinib (100 mg kg day^−1^) or/and terbinafine (20 mg kg day^−1^) for 6 weeks (**Figure** **6**A). Analysis of c‐PARP expression and TUNEL assays revealed that terbinafine significantly inhibited crizotinib‐induced cell apoptosis (Figure 6B,C). Moreover, combination treatment with terbinafine reversed the increases in the levels of serum liver injury biomarkers, including ALT, AST, LDH, and ALP (Figure 6D). H&E staining and Oil Red O staining jointly revealed that terbinafine markedly relieved the massive infiltration of inflammatory cells caused by crizotinib and the accumulation of lipid droplets (Figure 6E,F). The abnormal metabolic process was also ameliorated by terbinafine, as evidenced by alterations in liver TC and TG levels (Figure 6G). Notably, a previous study demonstrated that SQLE functions as a monooxygenase that uses an oxygen atom from O_2_ to oxidize squalene and reduces the other oxygen atom by reducing equivalents from NADPH (the reduced form of NADP^+^) to generate NADP^+^. The exhaustion of NADPH by SQLE disturbs redox homeostasis, which results in oxidative stress.^[^ ^26b^ ^]^ By combining terbinafine with crizotinib, the increased NADP^+^ and decreased NADPH levels could be partially reversed, thus resulting in the activation of oxidative stress (Figure 6H,I). Taken together, these data prove that the SQLE enzymatic activity inhibitor terbinafine demonstrates potential as a treatment for crizotinib‐induced liver toxicity.

## 3 Discussion

**Figure 7:**
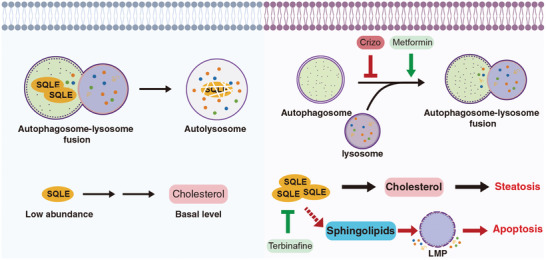
Schematic diagrams of the mechanism of crizotinib hepatotoxicity. Crizotinib prevents autophagosome‐lysosome fusion and induces the excessive accumulation of SQLE. The accumulated SQLE, through its enzymatic activity, leads to an increase in cholesterol and subsequently causes steatosis in liver hepatocytes. Moreover, when exposed to crizotinib, SQLE can also disturb sphingolipid metabolism, resulting in the accumulation of ceramide and sphingosine within lysosomes and thereby inducing cell apoptosis. Notably, autophagy activators, such as metformin, or SQLE enzymatic inhibitors, like terbinafine, possess potential clinical use for alleviating crizotinib‐induced hepatotoxicity by restoring the level or activity of SQLE.

In this study, we first revealed that cholesterol metabolism anomalies, cell apoptosis and autophagy blockade are the major causes of crizotinib‐induced hepatotoxicity. Furthermore, our findings revealed that the accumulation of SQLE caused by the inhibition of autophagosome‐lysosome fusion is associated with cholesterol metabolic abnormalities and apoptosis. More importantly, we revealed the novel finding that the alterations in SQLE levels have an impact on sphingolipid regulation and the induction of cell apoptosis. Both the reduction in SQLE protein levels via the autophagy activator metformin and the direct inhibition of enzymatic activity via terbinafine can significantly ameliorate crizotinib‐induced hepatotoxicity, thereby offering a clinical intervention strategy for the safer use of crizotinib (**Figure** **7**).

Cholesterol is essential for maintaining basic biological functions, such as the metabolism of synthetic cell membranes, steroid hormones, vitamin D3, and bile acids.^[^ ^32^ ^]^ Cholesterol homeostasis is elaborately regulated and involves dietary intake, biosynthesis in the liver, biliary excretion and peripheral tissue uptake.^[^ ^11a^ ^]^ The liver is known as the major organ that is primarily responsible for cholesterol synthesis, which indicates that drug‐induced liver injury is susceptible to inducing cholesterol metabolic anomalies, thereby disrupting the balance with respect to systemic cholesterol. In this study, we demonstrated that crizotinib‐induced perturbance of cholesterol homeostasis contributed to its hepatotoxicity via the excessive accumulation of the cholesterol biosynthesis enzyme SQLE. A previous study demonstrated that a high‐cholesterol diet in *Alms1* mutant mice, which are characterized by a metabolic profile similar to that of NASH, led to hepatocyte apoptosis, macrophage recruitment, and fibrosis but without ballooning degeneration.^[^ ^33^ ^]^ In our in vivo models, cell apoptosis and inflammatory cell infiltration were the most obvious pathological changes, which is consistent with high cholesterol‐induced metabolic dysfunction‐associated fatty liver disease (MAFLD). Notably, ballooning degeneration, which is the classical indicator of MAFLD, is mostly caused by the excessive accumulation of triglycerides.^[^ ^34^ ^]^ However, we discovered that the degree of TG upregulation after crizotinib treatment was mild, which suggests that the absence of ballooning degeneration was a reasonable finding. Our study revealed that the major contributor to elevated liver cholesterol content included increased cholesterol biosynthesis due to the overregulation of SQLE. Nevertheless, based on research about dysregulation of cholesterol metabolism caused by MAFLD, hydroxymethylglutaryl‐CoA reductase (HMGCR) and sterol regulatory element‐binding protein 2 (SREBP2) are mostly responsible for the disturbance of cholesterol homeostasis and thereby abnormally increase cholesterol content.^[^ ^35^ ^]^ These differences may result from the different initial mechanisms by which crizotinib inhibited the autophagic degradation of SQLE compared with a high‐fat diet, which directly upregulated HMGCR at the transcriptional level via SREBP2. Hence, our data emphasized the pivotal role of SQLE in cholesterol metabolism (at least in drug‐induced liver injury).

SQLE is considered to be the second rate‐limiting enzyme in cholesterol synthesis, and its inhibition can effectively reduce cholesterol synthesis.^[^ ^36^ ^]^ The reaction involving the conversion of squalene (which is catalyzed by SQLE) requires the involvement of the requisite transfer partner, NADPH‐cytochrome P450 reductase. In our study, we observed an abnormal increase in SQLE, which was accompanied by the downregulation of NADPH and increased NAD^+^, thus indicating the overactivation of SQLE enzymatic activity. Notably, SQLE has been reported to be upregulated in multiple cancers. Li et al. demonstrated that SQLE is highly expressed in colorectal cancer and that SQLE accelerates carcinogenesis by inducing the cell cycle and suppressing apoptosis.^[^ ^15a^ ^]^ Additionally, Ye et al. revealed the overactivation of SQLE in glioblastoma, which rewired cholesterol metabolism and favored the growth of glioblastoma.^[^ ^14^ ^]^ However, in crizotinib‐induced hepatotoxicity, our research revealed that elevated SQLE provoked cell apoptosis in normal hepatocytes both in vivo and in vitro. These conflicting findings may be due to the separate metabolic alterations occurring between cancer cells and normal cells. Moreover, the upregulation of SQLE causes high cholesterol levels, which arrest cell growth in normal tissue cells^[^ ^24^, ^37^ ^]^ but initiate and promote cancer progression.^[^ ^38^ ^]^ Regarding the functional mode of accumulated SQLE in autophagosome, we hypothesize that SQLE may be located on their membranes given that autophagosome originate from endoplasmic reticulum,^[^ ^39^ ^]^ where SQLE catalyzes cholesterol de novo synthesis.^[^ ^40^ ^]^ Alternatively, the autophagosome may still provide a biologically suitable environment for SQLE to function, leading to local accumulation of cholesterol.

Sphingolipids play important roles in cellular signaling transduction, with the two central bioactive lipids ceramide and sphingosine‐1‐phosphate exerting opposing effects on cell death.^[^ ^29^ ^]^ In our research, we demonstrated that ceramide and its metabolite sphingosine were upregulated in the context of crizotinib and that these changes could be reversed by *Sqle* knockdown. Notably, crizotinib induced the downregulation of sphingosine‐1‐phosphate, as demonstrated by our metabolomics data uploaded to the OMIX database (OMIX007371). The production of ceramide is catalyzed by acid sphingomyelinase and further transversion to sphingosine by lysosomal acidic ceramidase.^[^ ^41^ ^]^ The regulatory effect of SQLE on ceramide or sphingosine was assessed in our study; however, the molecular mechanism by which SQLE influences the metabolism of these molecules has not yet been clarified. Thus, whether SQLE influences with acid sphingomyelinase or acidic ceramidase (thereby regulating the cellular content of ceramide or sphingosine) warrants further investigation. Moreover, sphingosine reportedly appears early in the apoptotic pathway and acts either solely or together with ceramide.^[^ ^42^ ^]^ In our study, the increase in ceramide and sphingosine seemed to occur concomitantly, and we believe that ceramide and sphingosine are the same contributors to apoptosis, which aligns with the overall metabolic changes that were observed after treatment with crizotinib.

Metformin is a pharmacological activator of autophagy, and its mechanism is generally thought to be the phosphorylation of AMPK (Thr172).^[^ ^43^ ^]^ But our previous study demonstrated that metformin reversed crizotinib‐induced cardiotoxicity by activating AMPK (Ser485/491) and restoring the blockade of autophagosome‐lysosome fusion.^[^ ^3^, ^44^ ^]^ In the present study, we demonstrated that metformin interferes with crizotinib hepatotoxicity via the restoration of autophagic degradation in SQLE and whose mechanism could also be attributed to the activation of p‐AMPK (Ser485/491), thereby supporting the phosphorylated site of AMPK at Ser485/491 is another pharmacological target of metformin. Moreover, metformin has been reported to exert a significant effect on systemic metabolism;^[^ ^45^ ^]^ specifically, metformin can inhibit cholesterol metabolism in multiple tissues.^[^ ^46^ ^]^ Furthermore, in the mouse model, the combination with metformin showed better improvement in the upregulated cholesterol, TG and LDL‐C when compared with suppression of SQLE via shRNA. Hence, the intervention effect of metformin may be due not only to its ability to remedy excessively elevated SQLE but also to its independent effect on cholesterol metabolism. Another prospective intervention strategy for crizotinib hepatotoxicity that was demonstrated in this study included terbinafine. Terbinafine, which is a canonical SQLE inhibitor, has been approved by the FDA for treating antifungal infections.^[^ ^15a^ ^]^ The concentration of terbinafine that was used in our research was confirmed based on a previous study on colorectal cancer, which demonstrated its safety and effectiveness.^[^ ^47^ ^]^ We revealed that terbinafine effectively interferes with crizotinib‐induced hepatotoxicity by inhibiting the enzymatic activity of SQLE.

Crizotinib serves as the first‐line treatment for ALK‐positive NSCLC; additionally, according to existing research, lung cancer is the second most common cancer and the most lethal cancer.^[^ ^48^ ^]^ The outstanding efficacy of crizotinib results in ALK‐positive NSCLC being considered a manageable chronic disease. However, various toxicities (such as hepatotoxicity, cardiotoxicity, pulmonary toxicity etc.) have significantly limited the clinical use of crizotinib. Our research revealed that crizotinib‐induced autophagy arrest leading to the upregulation of SQLE was the major cause of hepatotoxicity. Notably, the mechanism of autophagy inhibition involves both cardiotoxicity and pulmonary toxicity,^[^ ^3^, ^44^ ^]^ thus indicating the potential involvement of pharmacological targets in its hepatotoxicity. Comparative toxicity mechanism analyses with other known ALK inhibitors (including brigatinib, ensartinib, ceritinib and lorlatinib) have revealed all ALK inhibitors except ceritinib downregulated p‐AMPK (Ser485/491). The varying inhibition of p‐AMPK (Ser485/491) should consider the inhibition of different downstream process in response to receptor inhibition, as it has been reported several kinases, such as AKT, ERK, S6K and PKD1, could regulate p‐AMPK (Ser485/491).^[^ ^49^ ^]^ The LC3‐II levels were increased after treatment with ALK inhibitors, indicating the possible occurrence of autophagosome‐lysosome fusion blockage. Upon examination, it appears that there is no discernible positive correlation between the magnitude of AMPK inhibition and the accumulation of LC3‐II levels, a phenomenon that may be attributable to the inherent capacity of the inhibitor to facilitate autophagosome formation. Our findings revealed that crizotinib and lorlatinib increased the SQLE protein level to a certain extent, while other inhibitors did not produce a significant effect, suggesting that the observed mechanism is quite unique among ALK inhibitors (Figure S14A, Supporting Information). Notably, the result regarding lorlatinib aligns with clinical observations, where patients undergoing treatment with lorlatinib may experience severe lipid metabolism abnormalities, such as hypercholesterolemia and hypertriglyceridemia.^[^ ^50^ ^]^ Consequently, the intervention strategies employed for managing crizotinib‐induced hepatotoxicity, which include the use of metformin or terbinafine, may potentially be extended to the hepatoprotective context of lorlatinib treatment as well, though this requires further investigation.

Last but not least, the key toxicity initiation mechanism of crizotinib is quite different from that of other drugs known to induce hepatotoxicity through lipid metabolism disturbance. As summarized in a review, the interplay between a chemical and a nuclear receptor or transcription factors was the primary and pivotal event for initiating drug‐induced steatosis in regarding most drugs documented in AOP‐Wiki.^[^ ^2b^ ^]^ However, when exposed to crizotinib, the initial event leading to lipid metabolism disturbance are the blockage of autophagosome‐lysosome fusion and the subsequent SQLE accumulation due to autophagic degradation inhibition. The above comparison indicates that protein stability dysregulation is the cause of crizotinib lipid metabolism disturbance while abnormal transcriptional alterations of lipid metabolism genes are present in the treatment with other drugs initially. Considering that it is notoriously difficult to directly target transcriptional factors,^[^ ^51^ ^]^ the intervention strategies for most other drug‐induced steatosis mainly focuses on alleviating lipid accumulation‐induced hepatic oxidative stress and cell death, thereby ignoring impediment of toxicity initiation events. In contrast, targeting toxicity initiation event in crizotinib represents as more specific and effective toxicity intervention strategy.

In summary, we revealed that accumulated SQLE is a novel contributor to metabolite‐induced hepatotoxicity, which disturbs cholesterol/sphingolipid homeostasis, thus further resulting in both steatosis and apoptosis. The targeting of SQLE offers potential therapeutic promise for the treatment of metabolism anomaly‐related diseases, thereby broadening the clinical range span of SQLE inhibitors.

## 4 Experimental Section

### Animal Study and Approval

6–8 weeks male C57BL/6J mice were purchased from the Zhejiang Vital River Laboratory Animal Technology Co., Ltd. (Jiaxing, China). All mice procedures were performed according to the Institutional Animal Care and Use Committee (IACUC) protocol of Innovation Institute for Artificial Intelligence in Medicine of Zhejiang University attached by ethical number (DW22072901, DW202303291056, and DW202309051608). The mice were housed in the animal facilities with a 12 h light dark cycle, food and water available ad libitum. Before performing in vivo experiment, 1 week period was provided to allow the animals to adapt to the laboratory environment. Crizotinib (T1661, Topscience, Shanghai, China), metformin (MB1927, Meilunbio, Dalian, China) and terbinafine (MB1239, Meilunbio, Dalian, China) were dissolved in 20% cyclodextrin (H108813, Aladdin, Shanghai, China) to obtain stock. In the study of crizotinib hepatotoxicity, the mice were treated with 20% cyclodextrin or 100 mg kg^−1^ crizotinib daily via intragastric administration for 6 weeks; in the study of *Sqle* knockdown effect on crizotinib hepatotoxicity, the mice were injected with AAV8‐TBG promoter‐sh*Sqle* or AAV8‐TBG promoter‐*N.C* for the inhibition of SQLE followed by the treatment of 20% cyclodextrin or 100 mg kg^−1^ crizotinib daily via intragastric administration for 6 weeks; in the study of metformin precaution effects, the mice were treated with 20% cyclodextrin, 100 mg kg^−1^ crizotinib, 200 mg kg^−1^ metformin or crizotinib plus metformin daily via intragastric administration for 6 weeks; in the study of terbinafine precaution effects, the mice were treated with 20% cyclodextrin, 100 mg kg^−1^ crizotinib, 20 mg kg^−1^ terbinafine or crizotinib plus terbinafine daily via intragastric administration for 6 weeks.

Specific knockdown of *Sqle* in mouse hepatocytes is achieved by adeno‐ associated virus (AAV). A liver‐specific adeno‐associated virus serotype 8 (AAV8) carrying with TBG promoter and shRNA targeting for *Sqle* was constructed and packaged by WZ Biosciences Inc (Jinan, China). The AAV8‐TBG promoter‐*N.C* and AAV8‐TBG promoter‐sh*Sqle* were injected into C57BL/6J mice through tail vein followed by 3 weeks conveying. Then, the mice were treated with 20% cyclodextrin or 100 mg kg^−1^ crizotinib by gavage daily for 6 weeks.

### Blood Biochemistry Analysis

The blood was collected into the 1.5 mL collection tubes by bleeding mice from the retro‐orbital plexus under isoflurane anesthesia. Then, the blood samples were set aside at room temperature for more than one hour. Subsequently, the whole blood samples were centrifuged at 4000 rpm for 10 min to collect the serum for the detection of alanine aminotransferase (ALT), aspartate aminotransferase (AST), alkaline phosphatase (ALP) and lactate dehydrogenase (LDH).

### Histopathological and Immunohistochemical Analysis

Mice were sacrificed to harvest the liver and the liver was divided into three parts. One of them was quickly frozen in liquid nitrogen and stored at −80 °C when another one was fixed with 4% paraformaldehyde (PFA) (P6148, Sigma–Aldrich, St. Louis, USA). The last one was fixed in 10% phosphate‐buffered formalin (F8775, Sigma–Aldrich, St. Louis, USA) (pH = 7.4) and embedded in paraffin before cut into 4 µm slices. The tissue sections were processed and stained with hematoxylin and eosin (H&E) according to standard protocols. For immunohistochemical analysis, liver sections were incubated with the following primary antibody: SQLE (A2428, Abclonal, Wuhan, China). Finally, all processed slides were recorded and analyzed on pathological section scanner (HS6, SUNNY INSTRUMENT CO., LTD., Ningbo, China).

### TUNEL Assay

One Step TUNEL apoptosis assay kit (C1088, Beyotime, Shanghai, China) was utilized to detect apoptosis events in liver section according to manufacturer's instruction. Briefly, the tissue sections were pretreated with Proteinase K (ST532, Beyotime, Shanghai, China) working solution for 30 min at 37 °C in a humidified chamber after dewaxing and rehydration. Then, TUNEL detection solution was added on the slides and incubated with tissues samples for 60 min at 37 °C protecting from light. Finally, nuclei were stained with 4′,6‐diamidino‐2‐phenylindole (DAPI) (D212, Dojindo, Kumamoto, Japan) and the TUNEL signals were observed with fluorescence microscope (IX81‐FV1000, Olympus, Tokyo, Japan).

### Dihydroethidium Assay

For tissue dihydroethidium (DHE) staining, liver tissues were fixed with 4% paraformaldehyde overnight and then dehydrated with 30% sucrose buffer (10 021 418, Sinopharm Chemical Reagent, shanghai, Chian). After that, the tissues were embedded in Optimal Cutting Temperature Compound (4583, Sakura Finetek, Nagano‐Pref, Japan) and followed by cutting into 5 µm frozen sections. ROS production in liver tissues was detected with DHE probes (S0063, Beyotime, Shanghai, China). DHE was dissolved in dimethyl sulfoxide (DMSO) at a stock concentration of 5 mM and diluted with Phosphate Buffered Saline (PBS) to 1 µM before using. The liver slides were incubated with DHE in a light‐protected humidified box at 37 °C for 30 min. Nuclei were stained with DAPI and imaged with a fluorescence microscope.

### Oil Red O Staining

For Oil Red O staining, adding 3 parts of Oil Red O stock solution (O1391, Sigma–Aldrich, St. Louis, USA) to one part of distilled water and mix them well for the further filter through a 45 µm filter to acquire Oil Red O working solution. The liver frozen slices were allowed to equilibrate for 10 min at room temperature before fixing with 4% PFA for 30 min. Then, the slices were immersed into 60% isopropanol for 1 min followed by incubation with Oil Red O working solution for 15 min at room temperature. At the end of incubation, rinse the slices with 60% isopropanol for 1 min and distilled water for 1 min orderly, the above procedure was repeated for 3 times to make the interstitium vivid. Finally, the slices were mounted with water‐soluble mounting medium and air‐dried overnight. A pathological section scanner was utilized to visualize the area of lipid accumulation.

### Liver Total Cholesterol, Triglyceride and Low‐Density Lipoprotein‐Cholesterol Extraction and Analysis

For quantification of intrahepatic total cholesterol (TC), triglyceride (TG) and low‐density lipoprotein‐cholesterol (LDL‐C), tissue was minced into small pieces using scissors on ice and weighed. Then, tissue was homogenized with PBS whose volume was 9 times the tissue weight (mL/g). The homogenate was centrifuged at 2500 rpm for 10 min at 4 °C and the supernatant was collected in a separate tube. The TC, TG and LDL‐C levels were determined using the TC assay kit (A111‐1‐1), TG assay kit (A110‐1‐1) and LDL‐C assay kit (A113‐1‐1), respectively, and normalized to tissue protein concentration. The mentioned assay kits were purchased from Jiancheng Bioengineering Institute (Nanjing, China).

### Hepatic NADP^+^ and NADPH Extracted and Analysis

For detection of intrahepatic NADP^+^ and NADPH levels, liver was washed with pre‐cooled PBS and ≈10–30 mg tissue was minced into small pieces by scissors. The tissue pieces were mixed with extraction solution at the ratio of 200 µL solution per 10 mg tissue and homogenized completely. Subsequently, the homogenate was centrifuged at 12 000×g for 10 min at 4 °C and the supernatant was collected for further assessment of NADP^+^ or NADPH levels by using NADP^+^/NADPH Assay Kit with WST‐8 (S0179, Beyotime, Shanghai, China), and normalized to tissue protein concentration.

### Untargeted Metabolomics Analysis

The mice liver tissues were collected into the centrifuge tube and quickly frozen by liquid nitrogen immediately. Later, the samples were delivered to LC‐BIO Technologies (Hangzhou, China) for untargeted metabolomics analysis. The collected samples were thawed on ice when metabolites were extracted with lipid extraction buffer. All samples were acquired by the LC‐MS system based on machine orders. First, all chromatographic separations were performed using an ACQUITY UPLC System (Waters, Milford, USA). A Kinetex UPLC C18 column (100 mm * 2.1 mm, 100A, phenomenex, UK) was utilized for the reversed phase separation. The metabolites eluted from the column.A were detected by high‐resolution tandem mass spectrometer Q‐Exactive (Thermo Scientific, Waltham, USA). The Q‐Exactive was operated in both positive and negative ion modes. The acquired LC−MS raw data files were converted into mzXML format and then processed by the XCMS, CAMERA and metaX toolbox implemented with the R software. The online KEGG, HMDB database was applied to mark the metabolites by matching the exact molecular mass data (m/z) of samples with those from database. The intensity of peak data was further preprocessed by metaX. Those features that were detected in less than 50% of QC samples or 80% of biological samples were excluded and PCA was performed for outlier detection and batch effects evaluation using the pre‐processed dataset.

Student's *t*‐tests were conducted to detect differences inside metabolite concentrations between 2 phenotypes. The *P* value was adjusted for multiple tests using an FDR (Benjamini–Hochberg).

### Primary Hepatocyte Isolation

6‐week to 8‐week‐old male C57BL/6J mice were anesthetized for the isolation of primary hepatocyte. The inferior vena cava was cannulated and perfused with solution A containing EDTA and solution B containing collagenase IV sequentially for liver digestion. The portal vein was cut immediately upon appearance of white spots and repeated clamp once a minute during the infusion. At the end of digestion, the liver should be carefully dissected and transferred to dish followed by the liver rupture. The acquired suspension was filtered through a 70 µm strainer and then centrifuged at 50×g for 2 min at 4 °C. Next, the supernatant was aspirated and resuspended pellet with Percoll solution. Finally, the suspension was washed with plating media twice and plated for sequential study.

### Cell Culture and Treatment

Human primary hepatocytes were purchased from BioreclamationIVT (F00995‐P and M00995‐P, New York, USA), and the clinical characteristics of the donors were presented in Table S1 (Supporting Information). Human primary hepatocytes were cultured according to manufacturer's instructions. Briefly, human primary hepatocytes were thawed in INVITROGROTM CP Medium (S03316, BioreclamationIVT, New York, USA) supplemented with 10% fetal bovine serum (SH30396.03, Hyclone, Logan, USA) at 37 °C in a 5% CO_2_ incubator for 24 h and then replaced with INVITROTM HI Medium (Z99009, New York, USA).

HL‐7702 cells were purchased from Jennio Biological Technology (JNO‐048, Guangzhou, China) and were maintained in RPMI‐1640 (318 000, Gibco, California, USA). THLE‐2 cells were purchased from Wuhan Pricella Biotechnology Co., Ltd (CL‐0833, Wuhan, China) and were maintained in specialized cell culture medium for THLE‐2 (CM‐0833, Wuhan Pricella Biotechnology Co., Ltd., Wuhan, China). Human Embryonic Kidney 293 T cells (HEK293T) were kindly supplied by the Institute of Biochemistry and Cell Biology, Chinese Academy of Sciences (Shanghai, China) and were maintained in DMEM (128 000, Gibco, California, USA). AML12 cells were generously supplied by the Stem Cell Bank at the Chinese Academy of Sciences (Shanghai, China) and were maintained in DMEM/F‐12 (125 000, Gibco, California, USA), which was added with 10% ITS Media Supplement (C0341, Beyotime, Shanghai, China) and 1% 40 ng mL^−1^ Dexamethasone solution (ST1254, Beyotime, Shanghai, China). All of cell lines were grown supplement with 10% fetal bovine serum (SH30396.03, Hyclone, Logan, USA), 100 U ml^−1^ penicillin and 100 µg ml^−1^ streptomycin in a humidified atmosphere with 5% CO_2_ and 95% air at 37 °C.

Cycloheximide (S7418), bafilomycin A1 (S1413) and rapamycin (S1039) were purchased from Selleck Chemicals (Shanghai, China). MG‐132 (M8699) was purchased from Sigma–Aldrich (St. Louis, USA). Sphingosine (T5891) was purchased from Topscience (Shanghai, China). E64d (HY‐100229), brigatinib (HY‐12857), ceritinib (HY‐15656), ensartinib (HY‐103714) and lorlatinib (HY‐12215) were purchased from MedChemExpress (Shanghai, China). Throughout the experiments (if not stated otherwise), HL‐7702 cells were treated with 3 µM crizotinib for indicated time periods or with 0, 1.5, 3, 4.5 µµ crizotinib for 24 h. And HL‐7702 cells were treated with 3 µM brigatinib, 6 µM ceritinib, 3 µM crizotinib, 3 µM ensartinib and 4.5 µM lorlatinib for 24 h. In selected experiments, 1.5 mM metformin, 10 µg mL^−1^ cycloheximide, 20 µM MG‐132, 1 nM bafilomycin A1, 1 µM rapamycin, 2.5 µM sphingosine and 10 µM E64d were added and incubation for 24 h. Moreover, in some cases, human primary hepatocytes, mouse primary hepatocytes, THLE‐2 cells and AML12 cells were treated with 0, 1.5, 3, 4.5 µµ crizotinib for 24 h. And human primary hepatocytes were treated 3 µµ crizotinib with 1.5 mM metformin for 24 h in certain circumstance. In other cases, 10 µM MG‐132 was added 6 h before HEK293T cells collection.

### BODIPY Staining

Intracellular neutral lipid content was visualized and quantified by using BODIPY 493/503 fluorescent probes (MX5403, Maokang Biotechnology Co., Ltd., Shanghai, China) according to the manufacturer's instruction. Briefly, cells were seeded in 12‐well plates (8 × 10^4^ cells well^−1^ for HL‐7702 cells) and left for 24 h free growth. Then, cells were treated with 0 or 3 µM crizotinib for 24 h. At the end of treatment period, the culture medium was removed and the cells were rinsed with PBS carefully for twice. And the BODIPY stock solution was diluted by PBS to the final concentration at 2 µM. Then, cells were incubated with BODIPY staining solution in the dark for 15 min at 37 °C. Next, the supernatant was aspirated and the cells were washed with PBS for three times. The cell nuclei were indicated by Hoechst 33 342 (C0031, Solarbio Science & Technology Co., Ltd., Beijing, China) under the condition of 15 min incubation time at 37 °C in the dark. And wash the cells with PBS for 3 times followed by imaging with a fluorescence microscope.

### Transmission Electron Microscopic Analysis

Primary mouse hepatocytes were seeded into 12‐well plates and left for 24 h free growth. Then, the cells were treated with 0 or 3 µM crizotinib for 24 h, respectively and fixed in 2.5% glutaraldehyde solution (G5882, Sigma–Aldrich, St. Louis, USA), post‐fixed in 1% osmic acid (419 494, Sigma–Aldrich, St. Louis, USA), dehydrated, embedded, sliced at a thickness of 1 µm and stained with uranyl acetate (21447–25, Polysciences, Warrington, USA) and lead citrate (HD17810, HEDE BIOTECHNOLOGY Co., LTD., Beijing, China). The intracellular ultrastructure was imaged by PHILIPS TECNAI 10 electron microscope (TECNAI 10, Thermo Fisher Scientific, Waltham, USA).

### Autophagic Flux Measurement

Ad‐mCherry‐GFP‐LC3B (AD202001, WZ Biosciences Inc., Jinan, China) was utilized to visualize intracellular autophagic flux. Briefly, HL‐7702 cells were seeded into Nunc Lab‐Tek II Chamber Slide (154 534, Thermo Fisher Scientific, Waltham, USA) and infected with Ad‐mCherry‐GFP‐LC3B. After virus transfection for 6 h, the supernatant was replaced by fresh culture medium and cells were left for 12 h free growth. Then, cells were treated with 3 µM crizotinib or/and 1.5 mM metformin for 24 h. At the end of incubation, cells were washed with PBS twice and fixed with 4% PFA for 30 min at room temperature. And cells were then washed with PBS for 3 times and permeabilized by 0.1% Triton X‐100 solution for 30 min at 4 °C. After that, nuclei were stained with DAPI and sealed by antifade mounting medium. Finally, the images were captured by a fluorescence microscope. Notably, the process after virus infection should be protected from light.

### TMT‐Based Quantitative Analysis of Proteome

An integrated method involving TMT labeling and LC‐MS/MS to quantify the dynamic changes of the whole proteome in human hepatocytes was performed by LUMINGBIO (Shanghai, China). In brief, HL‐7702 cells were treated with 0 or 3 µM crizotinib for 24 h. The quantitative process encompassed protein extraction, SDS‐PAGE quality testing, trypsin digestion, TMT labeling, HPLC fractionation, LC‐MS/MS analysis, database annotation and bioinformatic analysis. The fold change cutoff was set when proteins with quantitative ratios above 1.5 or below 1/1.5 were deemed significant.

### RNA Interference

HL‐7702 cells were seeded into 12‐well plate (5 × 10^4^ cells well^−1^) and grown to ≈50% confluence. Then, the cells were transfected with control siRNA (negative control) or siRNA against *APOB*, *SQLE*, *CYP51A1*, *HMGCS1*, *FDFT1* or *MVK* using siRNA Transfection Reagent (409‐10, Polyplus‐transfection, Illkirch, France) according to the manufacturer's instructions. The following siRNAs were purchased from GenePharma (Shanghai, China).

siRNA targeting *APOB*, 5′‐UCCCCGGUCAGCGGAUAGU‐3′; siRNA targeting *SQLE*, 5′‐AACAUGAUAACCACCCGGC‐3′; siRNA targeting *CYP51A1*, 5′‐CAACUACUAGUGCUUGGAU‐3′; siRNA targeting *HMGCS1*, 5′‐UGUGCUAGAACAGAUGCAA‐3′; siRNA targeting *FDFT1*, 5′‐CGGCCAAGUCAAUAUUCUC‐3′; siRNA targeting *MVK*, 5′‐CACGGGAACCCCUCCGGAG‐3′; siRNA targeting negative control, 5′‐UUCUCCGAACGUGUCACGUdTdT‐3′.

### Flow Cytometric Analysis

For Annexin V‐PI staining, HL‐7702 cells were seeded in 12‐well plates (8 × 10^4^ cells well^−1^) and left for 24 h of free growth. Then, cells were treated as indicated. At the end of the incubation period, cells were harvested and washed with PBS. The Annexin V‐FITC/PI Apoptosis Kit (AP101, MultiSciences (Lianke) Biotech, Hangzhou, China) was applied to detect the apoptotic rate by using a CytoFLEX cytometer (B53015, Beckman Coulter, Suzhou, China) according to manufacturer's instruction.

### In Vitro TC Analysis

For in vitro TC analysis, human primary hepatocytes, mouse primary hepatocytes and HL‐7702 cells were seeded into 6‐well plates (2 × 10^5^ cells well^−1^) and left for 24 h of free growth followed by the indicated treatment. At the end of the incubation, the culture medium was removed and the cells were washed with PBS for once. Then, the cells were collected into BeyoLysis Buffer A for metabolic assay and homogenized by glass homogenizer accompanied by centrifuging at 12 000×g for 5 min at 4 °C. After that, the supernatant was collected for the detection of intracellular TC levels by TC assay kit (S0211S, Beyotime, Shanghai, China) and the value was then normalized to cellular protein concentration.

### Western Blot Analysis

Protein lysates (30‐50 µg per sample) of cells or liver tissues were preparing using lysis buffer (composed of 150 mM NaCl, 50 mM Tris‐HCl, 2 mM EGTA, 2 mM EDTA, 25 mM β‐sodium glycerophosphate, 25 mM NaF, 0.3% Triton X‐100, 0.3% NP‐40, 0.3% leupeptin, 0.1% NaVO3 and 0.1% PMSF). The protein lysates were separated on 8%, 10% or 12% SDS‐PAGE gels, transferred to 0.45 µm PVDF membranes (IPVH00010, Millipore Corporation, Boston, USA) and blocked with skim milk. Incubation of primary antibodies, secondary antibodies and the ECL‐Plus Kit (P2300, NCM Biotech, Suzhou, China) was performed to detect the bands.

Primary antibodies directed against cleaved PARP (ET1608‐10) and cathepsin L (HA722063) were purchased from Huabio (Hangzhou, China). Primary antibodies against cleaved PARP (Asp214) (94 885), p‐AMPK (S485/491) (4185) and cathepsin B (31 718) were purchased from Cell Signaling Technology (Beverly, USA). Primary antibody against SQLE (sc‐271651) and AMPK (sc‐74461) was purchased from Santa Cruz Biotechnology (Dallas, USA). Primary antibody against LC3 (M152‐3) was purchased from MBL (Tokyo, Japan). Primary antibody against SQLE (A2428) was purchased from Abclonal (Wuhan, China). Primary antibody against cleaved PARP (ab32064) was purchased from Abcam (Cambridge, UK). Primary antibody against HA (db2603) and ACTB (db14040) were purchased from Diagbio (Hangzhou, China). Anti‐Flag antibody (AYC01‐100) was purchased from Shanghai Yoche Biotechnology (Shanghai, China). HRP‐labeled secondary antibodies (FDR007, FDM007) were purchased from Fude Biological Technology (Hangzhou, China).

### Immunofluorescence Assay

After different treatment, cultured cells grown on 96‐well plate were washed with PBS twice and fixed with 4% paraformaldehyde (P6148, Sigma‐Aldrich, St. Louis, USA) for 20 min. The cells were permeabilized with 0.1% Triton X‐100 in PBS for 10 min at 4 °C and blocked with 4% bovine serum albumin (B2064, Sigma–Aldrich, St. Louis, USA) in PBS for 30 min at 37 °C. Then, the primary antibodies were added and incubated with the cells at 4 °C overnight. At the end of incubation, the primary antibodies were removed and the cells were washed with PBS for twice. After that, cells were incubated with Alexa Fluor 488‐ or Alexa Fluor 594‐conjugated secondary antibodies (A32742, A21206, A11037 and A21202; 1:250, Thermo Fisher Scientific, Waltham, USA) at room temperature for 1 h. Nuclei were stained with 4′,6‐diamidino‐2‐phenylindole (DAPI) (D212, Dojindo, Kumamoto, Japan) for 5 min and then imaged with a fluorescence microscope. The following primary antibodies have been used: Primary antibodies against SQLE (A2428) and TFEB (A7311) were purchased from Abclonal (Wuhan, China). Primary antibodies against LC3B (sc‐376404) and LAMP1 (sc‐20011) were purchased from Santa Cruz Biotechnology (Dallas, USA).

### Plasmid Construction and Transfection

pcDNA3.0‐SQLE was generated by restriction digestion of the pcDNA3.0 vector plasmids with KpnI and BamHI, followed by ligations. KpnI‐HF (R3142L), BamHI‐HF (R3136L) and T4 DNA ligase (M0202S) were purchased from New England Biolabs (Ipswich, USA). A full‐length SQLE cDNA was obtained from hepatocytes by PCR with the following primers used: SQLE‐KpnI‐Forward: 5′‐AGTCAGGGTACCGCCACCATGTGGACTTTTCTGGGCAT‐3′ and SQLE‐ BamHI‐Reverse: 5′‐CTGACTGGATCCATGAACCATATACTTCATTT‐3′. The SQLE enzyme inactive mutant SQLE‐Y195F was synthesized from wild‐type SQLE by using a site‐directed mutagenesis kit (11003ES10, Yeasen Biotechnology, Shanghai, China). The primers used were listed as following: SQLE‐Y195F‐Forward: 5′‐TAAATGGTTTCATGATTCATGATCAGGAAAGCAAATCAGAGG‐3′ and SQLE‐Y195F‐Reverse: 5′‐AATCATGAAACCATTTACAACCTGGGCATCAAGACCTTCC‐3′. Cells were transfected with plasmids using the jetPRIME (101 000 046, Polyplus‐transfection, Illkirch, France) according to the manufacturer's suggestions. Briefly, cells were seeded into specific dish or plate and grown to ≈70% confluence. HL‐7702 cells were seeded into 12‐well plates (1 × 10^5^ cells well^−1^) and HEK293T cells were seeded into 6 cm culture dish (1.2 × 10^6^ cells dish^−1^). Then, the cells were transfected with transfection reagent based on the ratio (1 µg plasmids: 2 µL transfection reagent). After 6 h‐transfection, the medium was replaced by fresh complete medium. For 6 cm dish, 1 µg for per plasmid was transfected and for 12‐well plate, total 0.25–1 µg plasmids were used.

### Immunoprecipitation Assays

For the exogenous immunoprecipitation assay, cells were lysed in the 1% NP‐40 buffer (pH = 8.0): 25 mM Tris‐base, 150 mM NaCl, 10% glycerol and 1% NP‐40; protease inhibitor cocktail (5871, Cell Signaling Technology, Boston, USA) was added before use. The lysate (containing 500 µg of total protein) was centrifuged to collect supernatant and the protein concentration was detected. Subsequently, the supernatant (containing 500 µg of total protein) was immunoprecipitated with 20 µL anti‐Flag beads (SA042001, Smart‐Lifesciences, Changzhou, China) at 4 °C overnight. Then, the lysate was centrifuged to remove the supernatant and wash with 1% NP‐40 buffer, followed by western blot analysis.

### Quantitative Real Time Polymerase Chain Reaction

The procedures were described in a previous study.^[^ ^17^ ^]^ After indicated treatment, cells were harvested with the Trizol reagent (15 596 026, Thermo Scientific, Waltham, USA). Equal amounts of RNA were reverse transcribed into complementary DNA with the cDNA reverse transcription kit (AT311, TransGen Biotech, Beijing, China). Quantitative real time polymerase chain reaction (qPCR) was performed on a QuantStudio 3 Real‐Time PCR instrument (A28132, Applied Biosystems, Woodlands, Singapore) using the TB Green Premix Ex Taq (Tli RNaseH Plus) (RR420A, Takara, Tokyo, Japan). Fold changes in the expression of each gene were calculated by the comparative threshold cycle (Ct) method using the formula 2^−(ΔΔCt)^. Two independent biological samples were quantified in technical duplicates and expression values were normalized to housekeeper gene.

The primer sequences were as follows:

Human‐*APOB*‐Forward: 5′‐AGAGGACAGAGCCTTGGTGGAT‐3′; Human‐*APOB*‐Reverse: 5′‐CTGGACAAGGTCATACTCTGCC‐3′; Human‐*SQLE*‐Forward: 5′‐CTCCAAGTTCAGGAAAAGCCTGG‐3′; Human‐*SQLE*‐Reverse: 5′‐GAGAACTGGACTCGGGTTAGCT‐3′; Human‐ *CYP51A1*‐Forward: 5′‐CTCTTACCAGGTTGGCTGCCTT‐3′; Human‐ *CYP51A1*‐Reverse: 5′‐CTTGAGACTGTCTGCGTTTCTGG‐3′; Human‐*HMGCS1*‐Forward: 5′‐AAGTCACACAAGATGCTACACCG‐3′; Human‐*HMGCS1*‐Reverse: 5′‐TCAGCGAAGACATCTGGTGCCA‐3′; Human‐*FDFT1*‐Forward: 5′‐TGTGACCTCTGAACAGGAGTGG‐3′; Human‐*FDFT1*‐Reverse: 5′‐GCCCATAGAGTTGGCACGTTCT‐3′; Human‐*MVK*‐Forward: 5′‐GGAAAGTGGACCTCAGCTTACC‐3′; Human‐*MVK*‐Reverse: 5′‐GCTTCTCCACTTGCTCTGAGGT‐3′; Human‐*ACTB*‐Forward: 5′‐CACCATTGGCAATGAGCGGTTC‐3′; Human‐*ACTB*‐ Reverse: 5′‐AGGTCTTTGCGGATGTCCACGT‐3′.

Mouse‐*Fasn*‐Forward: 5′‐CCCCTCTGTTAATTGGCTCC‐3′; Mouse‐*Fasn*‐ Reverse: 5′‐TTGTGGAAGTGCAGGTTAGG‐3′; Mouse‐*Srebp1*‐Forward: 5′‐CACTTCTGGAGACATCGCAAAC‐3′; Mouse‐*Srebp1*‐ Reverse: 5′‐ATGGTAGACAACAGCCGCATC‐3′; Mouse‐*Actb*‐Forward: 5′‐GTGACGTTGACATCCGTAAAGA‐3′; Mouse‐*Actb*‐ Reverse: 5′‐GCCGGACTCATCGTACTCC‐3′.

### Detection of Lysosomal Intactness

The lysosomal intactness was assayed with the acridine orange (AO) uptake method. In brief, HL‐7702 cells were seeded into 96‐well plate (5 × 10^3^ cells well^−1^ if transfection with siRNA otherwise 8 × 10^3^ cells well^−1^) following by the indicated crizotinib treatment. Then, cells were washed with PBS for once and incubated with AO staining solution (dilution AO stock solution with culture medium to the final concentration of 10 µg mL^−1^) for 15 min at room temperature in the dark. At the end of incubation, the cells were observed under a fluorescence microscope. The AO stock solution (GL0329) was purchased from BioLab Technology Co., Ltd (Beijing, China).

### Visualization of Lysosomes

The lysosomes were visualized by using Lyso‐Tracker Red (C1046, Beyotime, Shanghai, China) according to the manufacturer's instruction. In brief, HL‐7702 cells were seeded into 96‐well plate (5 × 10^3^ cells well^−1^ if transfection with siRNA otherwise 8 × 10^3^ cells well^−1^) following by the indicated crizotinib treatment. Then, the culture medium was replaced by 37 °C prewarmed Lyso‐Tracker Red working solution (Dilution Lyso‐Tracker Red stock solution with HBSS to the final concentration of 50 nM). And the cell nuclei were indicated by incubation with Hoechst 33 342 simultaneously. The incubation step should last for 15 min at 37 °C in the dark followed by image capture under a fluorescence microscope.

### ELISA Assay

A human precoated sphingosine ELISA kit (A097799, Fusheng Industrial Co., Ltd., Shanghai, China) was conducted to detect intracellular sphingosine level according to manufacturer's instruction. In brief, HL‐7702 cells were seeded into 6‐well plate (1.5 × 10^5^ cells well^−1^ if transfection with siRNA otherwise 2 × 10^5^ cells well^−1^) following by crizotinib treatment. At the end of incubation period, cells were harvested and washed with PBS for twice. Then, 50 µL PBS were added for resuspension of cell pellet followed by quick‐frozen in liquid nitrogen and thawed on ice for 3 times. After that, the cell lysate was diluted 5‐fold by sample diluents and the diluted cell lysate as well as serial standard sphingosine were added at 50 µL well^−1^ in 96‐well plate precoated with antibody against sphingosine. Subsequently, 50 µL HRP‐labeled antigen was added to competitively bind with sphingosine antibody for 1 h at 37 °C. And the supernatant was then discarded and the plate was washed with 5 times with washing buffer thoroughly. Afterwards, chromophoric reagent and stop solution were added to each well successively (the interval period was 15 min) accompanied by detection of the absorbance at 450 nm wavelength within 15 min. The absorbance values were converted to specific concentration value in ng/L based on the logistic curve fitting of standard curve. The sphingosine concentration of different treatment group was further normalized to respective protein concentration.

### Statistical Analysis

Data were represented as the mean ± standard deviation (SD). Statistical comparisons between 2 groups were performed using a 2‐tailed Student's *t*‐test; comparisons between more than 2 groups were made using a one‐way analysis of variance (ANOVA) with follow‐up Dunnett T3 tests. *P* value of < 0.05 was considered statistically significant. Statistical analysis was performed using GraphPad Prism Version 8.0. All the statistical details of the experiments could be found in the figure legends.

## Conflict of Interest

The authors declare no conflict of interest.

## Author Contributions

H.Y. and X.H. contributed equally to this work. H.Y., X.H., and Y.Z. designed this project, analyzed the data; H.Y., X.H., Y.M., and S.Z. wrote and revised the draft manuscript; Y.Z., X.H., S.Z., and Y.C. initiated this project and performed most of the cellular and animal study experiments; X.H., Y.Z., and W.W. performed the proteomics, metabolomics experiments, and data analysis; X.C., X.Z., and X.W. offered clinical data support; Z.X., X.Y., B.Y., and Q.H. provided valuable suggestions and edited the manuscript; P.L. designed the project, edited the manuscript, and supervised the study. All authors read and approved the final manuscript.

## Supporting information



Supporting Information

## Data Availability

The data that support the findings of this study are openly available in OMIX at https://ngdc.cncb.ac.cn/omix/preview/ywmNwdy1, reference number 7371.
