# Supplementary material for: Disturbing Cholesterol/Sphingolipid Metabolism by Squalene Epoxidase Arises Crizotinib Hepatotoxicity
Source: Adv Sci (Weinh). 2025 Jan 21;12(14):2414923. doi: 10.1002/advs.202414923 (PMC11984922; doi:10.1002/advs.202414923)
Supplement: Supplementary file 1 — Supporting Information [file ADVS-12-2414923-s001.docx]

**Disturbing Cholesterol/Sphingolipid Metabolism by Squalene Epoxidase Arises Crizotinib Hepatotoxicity**

Hao Yan, PhD ^1#^, Xiangliang Huang, BS ^1#^, Yourong Zhou, MM ^1^, Yuan Mu, BS ^1^, Shaoyin Zhang, MM ^1^, Yashi Cao, BS ^1^, Wentong Wu, PhD ^1^, Zhifei Xu, PhD ^1^, Xueqin Chen, MD ^2^, Xiaochen Zhang MD ^3^, Xiaohong Wang, MD ^4^, Xiaochun Yang, PhD ^1^, Bo Yang, PhD ^1,5^, Qiaojun He, PhD ^1,5^, Peihua Luo, PhD ^1*^

^1^Center for Drug Safety Evaluation and Research of Zhejiang University, College of Pharmaceutical Sciences, Zhejiang University, Hangzhou 310058, China.

^2^Department of Thoracic Oncology, Hangzhou Cancer Hospital, Affiliated Hangzhou First People’s Hospital, School of Medicine, Westlake University, Hangzhou 310006, China.

^3^Department of Medical Oncology, The First Affiliated Hospital, Zhejiang University School of Medicine, Hangzhou 310003, China.

^4^Zhejiang Cancer Hospital, Hangzhou Institute of Medicine (HIM), Chinese Academy of Sciences, Hangzhou 310022, China.

^5^School of Medicine, Hangzhou City University, Hangzhou 310015, China

^#^ These authors contributed equally to this work.

**Supporting information includes 14 supplementary figures and legends as well as 1 supplementary table**

**Supplementary figures and legends**


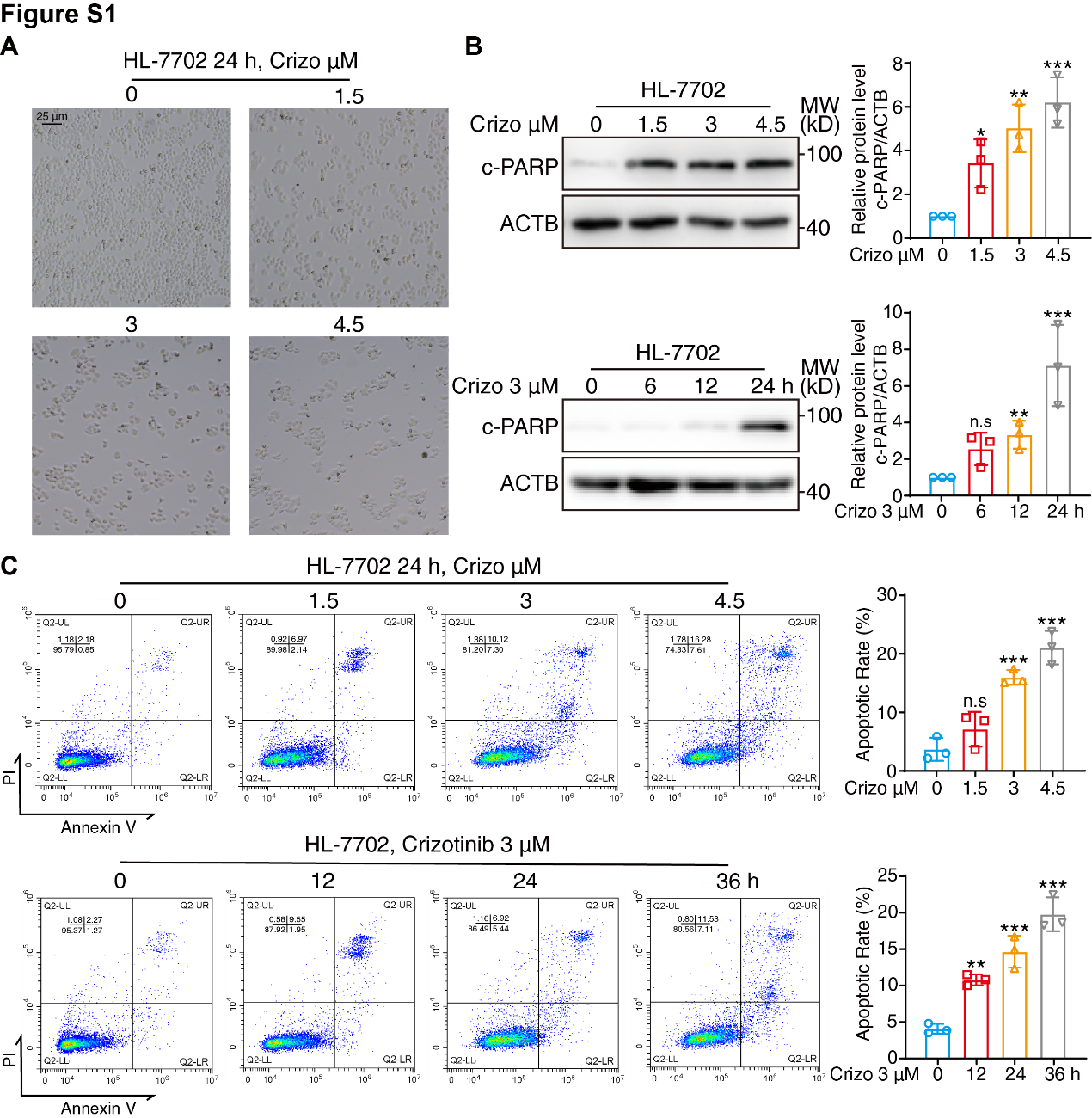


**Figure S1. Crizotinib induces apoptosis in HL-7702 cells. (A)** HL-7702 cells were treated with 0, 1.5, 3 and 4.5 μM crizotinib for 24 h. The number alteration of cells was observed under light microscope. Scale bar = 25 μm. **(B)** HL-7702 cells were treated with 0, 1.5, 3 and 4.5 μM crizotinib for 24 h or 3 μM crizotinib for 0, 6, 12 and 24 h. Relative expression of c-PARP was analyzed by western blot with ACTB as a loading control (n = 3 independent replicates). **(C)** HL-7702 cells were treated with 0, 1.5, 3 and 4.5 μM crizotinib for 24 h or 3 μM crizotinib for 0, 12, 24 and 36 h. The apoptotic rate was analyzed by flow cytometry combination with Annexin V/PI staining and representative images are shown (n = 3 independent replicates). The results are presented as the mean ± SD. The *P* value was calculated by one-way ANOVA (Dunnett’s multiple comparisons test). n.s = no significance; **P* < 0.05; ***P* < 0.01; ****P* < 0.001.


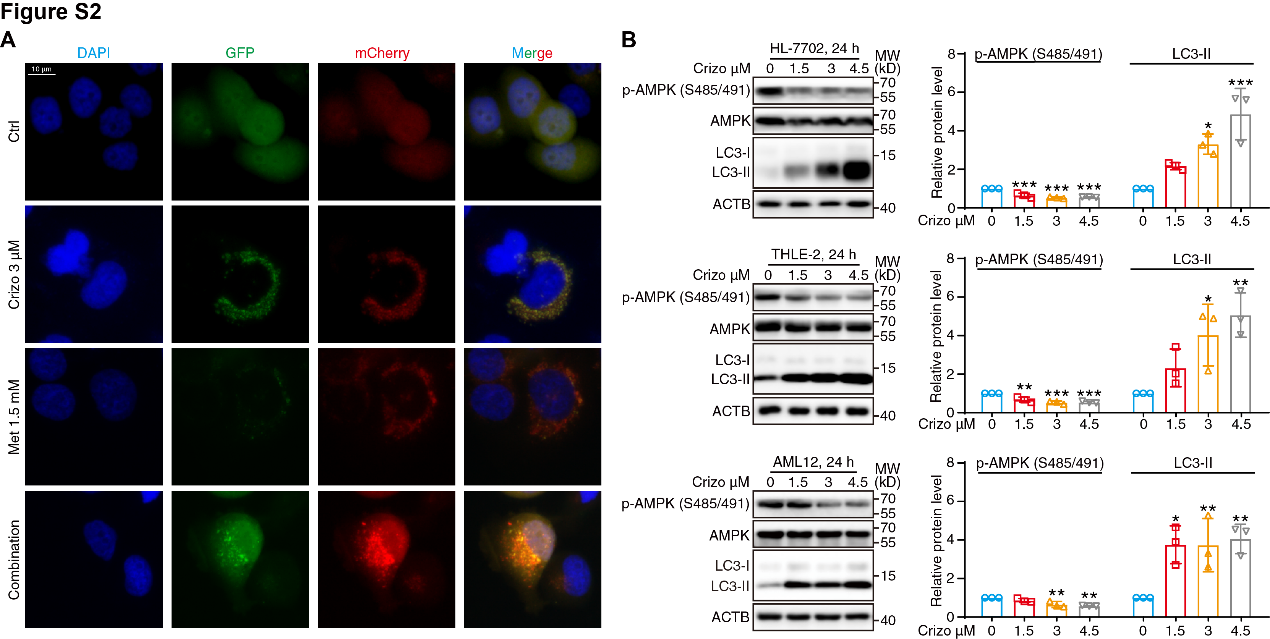


**Figure S2. Crizotinib induces blockade of autophagosome-lysosome fusion *in vitro*. (A)** HL-7702 cells were transfected with Ad-mCherry-GFP-LC3B followed by the treatment of 3 μM crizotinib with or without 1.5 mM metformin for 24 h. Representative confocal fluorescence images were presented with yellow puncta indicating autophagosomes and red puncta indicating autolysosomes within merged images. **(B)** HL-7702 cells, THLE-2 cells and AML12 cells were treated with 0, 1.5, 3 and 4.5 μM crizotinib for 24 h. Relative expressions of p-AMPK (S485/491), AMPK, and LC3 were analyzed by western blot with ACTB as a loading control (n = 3 independent replicates). Scale bar = 10 μm. The results are presented as the mean ± SD. The *P* value was calculated by one-way ANOVA (Dunnett’s multiple comparisons test). **P* < 0.05; ***P* < 0.01; ****P* < 0.001.


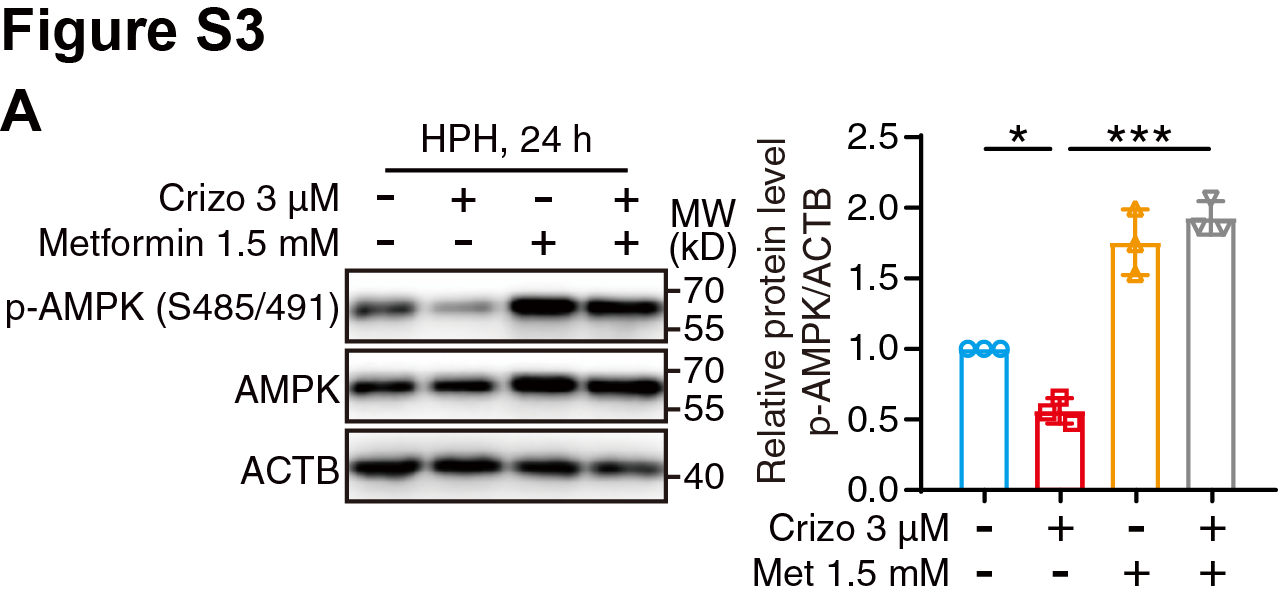


**Figure S3. Metformin relieves crizotinib-induced inhibition of p-AMPK (S485/491) in human primary hepatocytes. (A)** Human primary hepatocytes (HPH) from 3 donors were treated with 3 μM crizotinib with or without 1.5 mM metformin for 24 h. Relative expressions of p-AMPK (S485/491) and AMPK were analyzed by western blot with ACTB as a loading control. The results are presented as the mean ± SD. The *P* value was calculated by one-way ANOVA (Dunnett’s multiple comparisons test). **P* < 0.05; ****P* < 0.001.


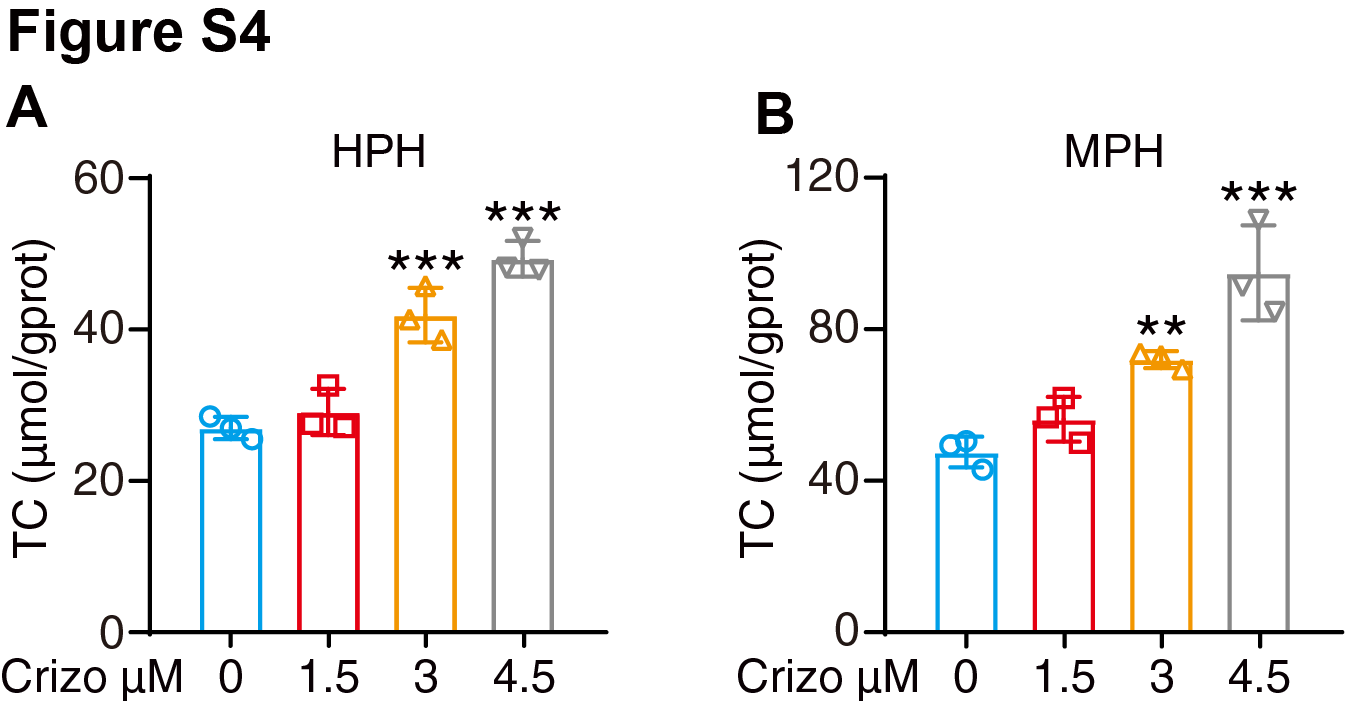


**Figure S4. Crizotinib increases intracellular cholesterol levels in human primary hepatocytes and mouse primary hepatocytes. (A)** Human primary hepatocytes (HPH) from 3 donors were treated with 0, 1.5, 3 and 4.5 μM crizotinib for 24 h. Total cholesterol levels within HPH were analyzed. (**B**) Mouse primary hepatocytes (MPH) were treated with 0, 1.5, 3 and 4.5 μM crizotinib for 24 h. Total cholesterol levels within MPH were analyzed (n = 3 independent replicates). The results are presented as the mean ± SD. The *P* value was calculated by one-way ANOVA (Dunnett’s multiple comparisons test). ***P* < 0.01; ****P* < 0.001.


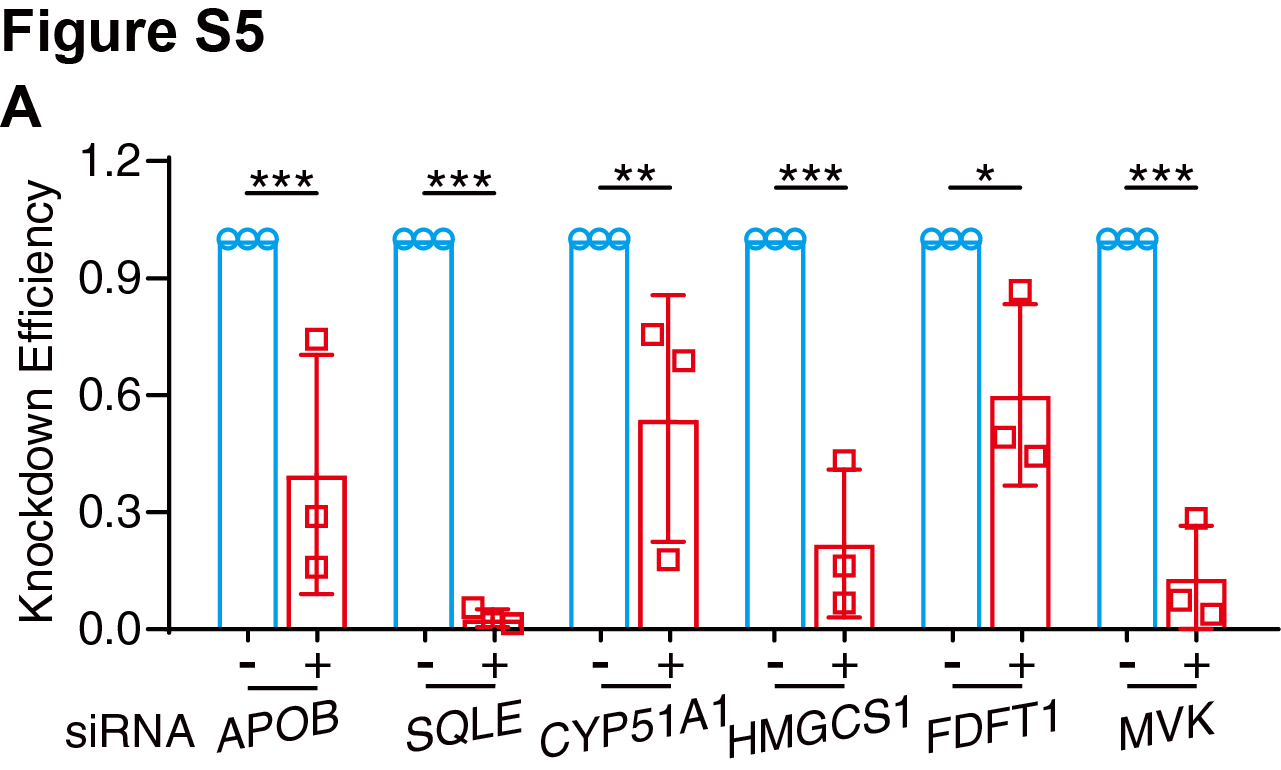


**Figure S5. The knockdown efficiency of siRNA targeting cholesterol biosynthesis pathway-related proteins in HL-7702 cells. (A)** HL-7702 cells were transfected with siRNA against *N.C*, *APOB*, *SQLE*, *CYP51A1*, *HMGCS1*, *FDFT1*, and *MVK* for 24 h. The knockdown efficiency of corresponding siRNA was analyzed by qPCR with ACTB as the internal reference (n = 3 independent replicates). The results are presented as the mean ± SD. The *P* value was calculated by Student’s *t* test (unpaired, two-tailed, 2 groups). **P* < 0.05; ***P* < 0.01; ****P* < 0.001.


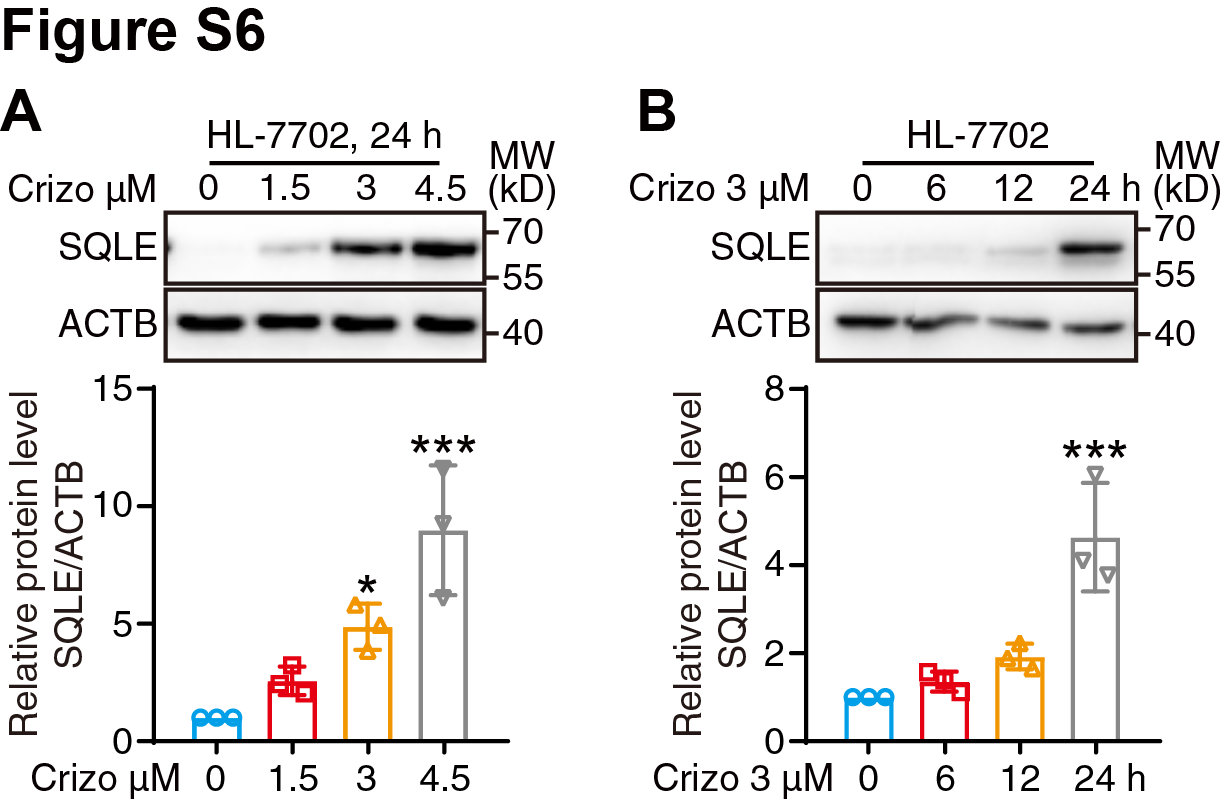


**Figure S6. Crizotinib induces SQLE accumulation in HL-7702 cells. (A, B)** HL-7702 cells were treated with crizotinib in a concentration- or time-dependent manner. Relative expression of SQLE was analyzed by western blot with ACTB as a loading control (n = 3 independent replicates). The results are presented as the mean ± SD. The *P* value was calculated by one-way ANOVA (Dunnett’s multiple comparisons test). **P* < 0.05; ****P* < 0.001.


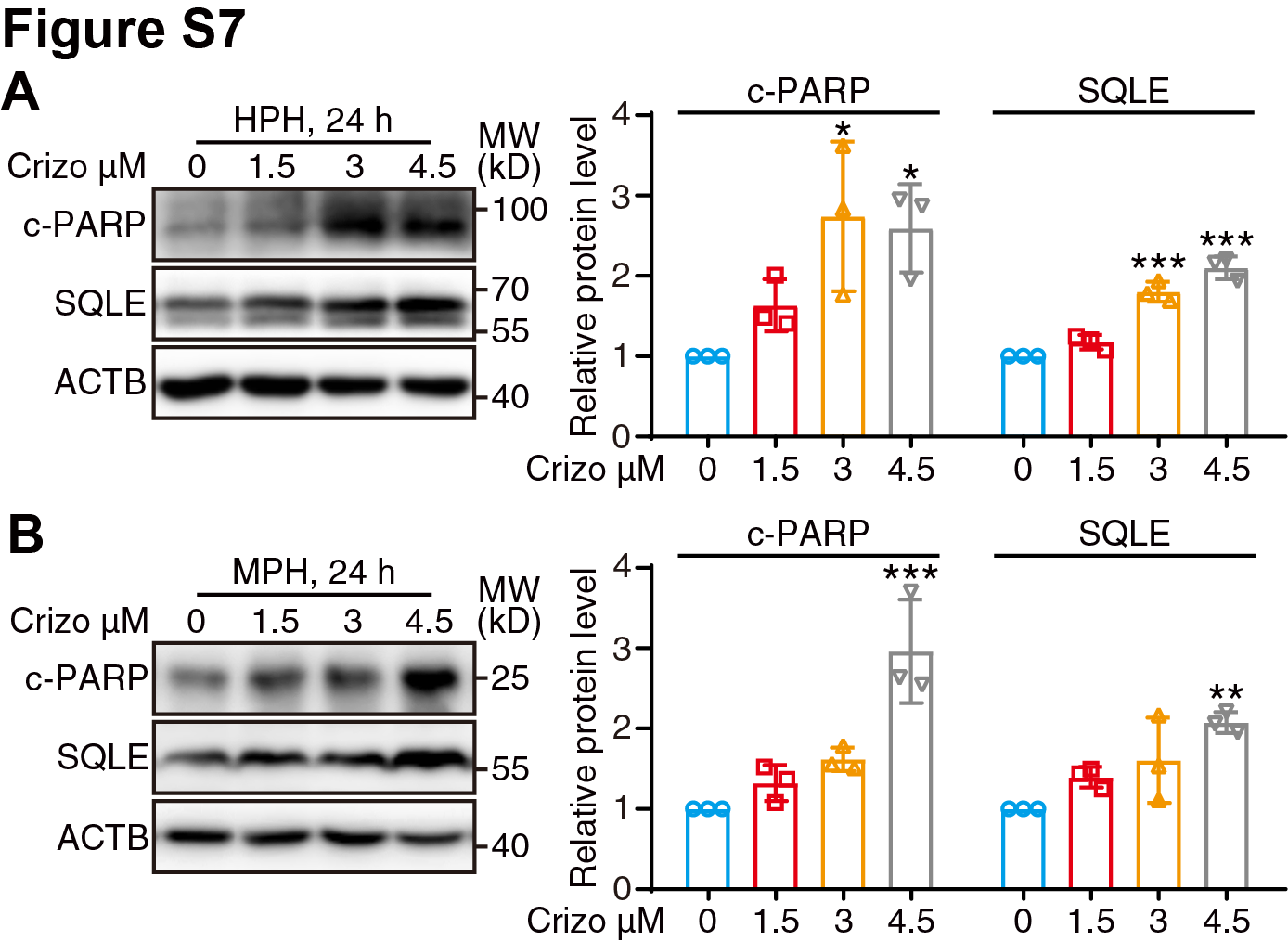


**Figure S7. Crizotinib induces SQLE accumulation and cell apoptosis in human primary hepatocytes and mouse primary hepatocytes. (A)** Human primary hepatocytes (HPH) from 3 donors were treated with 0, 1.5, 3 and 4.5 μM crizotinib for 24 h. Relative expressions of c-PARP and SQLE were analyzed by western blot with ACTB as a loading control. (**B**) Mouse primary hepatocytes (MPH) were treated with 0, 1.5, 3 and 4.5 μM crizotinib for 24 h. Relative expressions of c-PARP and SQLE were analyzed by western blot with ACTB as a loading control (n = 3 independent replicates). The results are presented as the mean ± SD. The *P* value was calculated by one-way ANOVA (Dunnett’s multiple comparisons test). **P* < 0.05; ***P* < 0.01; ****P* < 0.001.


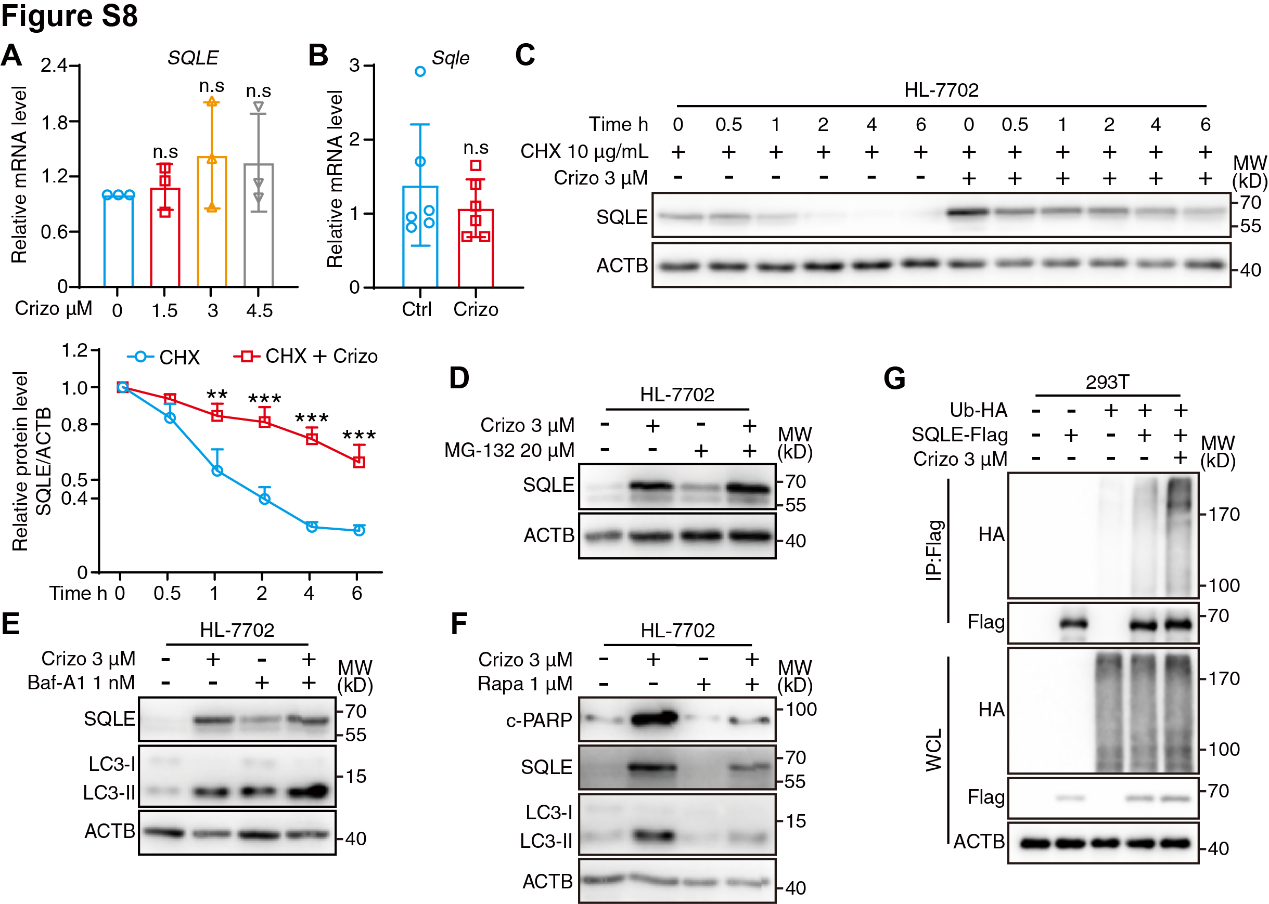


**Figure S8. Crizotinib-induced accumulation of SQLE was associated with autophagy inhibition in HL-7702 cells. (A)** HL-7702 cells were treated with 0, 1.5, 3 and 4.5 μM crizotinib for 24 h and the mRNA levels of *SQLE* were analyzed by qPCR with ACTB as an internal reference (n = 3 independent replicates). **(B)** The hepatic mRNA levels of *Sqle* from control group and crizotinib treatment group were analyzed by qPCR with ACTB as an internal reference (n = 6 per group). **(C)** HL-7702 cells were treated with 10 μg/mL CHX with or without 3 μM crizotinib for indicated period. Relative expression of SQLE was analyzed by western blot with ACTB as a loading control (n = 3 independent replicates). **(D)** HL-7702 cells were treated with 3 μM crizotinib for 24 h with or without 20 μM MG-132 added 6 h before harvest. Relative expression of SQLE was analyzed by western blot with ACTB as a loading control. **(E)** HL-7702 cells were treated with 3 μM crizotinib with or without 1 nM bafilomycin A1 for 24 h. Relative expressions of SQLE and LC3 were analyzed by western blot with ACTB as a loading control. **(F)** HL-7702 cells were treated with 3 μM crizotinib with or without 1 μM rapamycin for 24 h. Relative expressions of c-PARP, SQLE, and LC3 were analyzed by western blot with ACTB as a loading control. **(G)** HEK293T cells were transfected with SQLE-Flag and Ubiquitin-HA plasmid for 24 h, then HEK293T cells were treated as indicated. SQLE was immunoprecipitated by using anti-Flag beads and ubiquitylated SQLE was detected using an anti-HA antibody. The results are presented as the mean ± SD. The *P* value was calculated by Student’s *t* test (unpaired, two-tailed, 2 groups) for panel B and C or one-way ANOVA (Dunnett’s multiple comparisons test) for panel A. n.s = no significance; ***P* < 0.01; ****P* < 0.001.


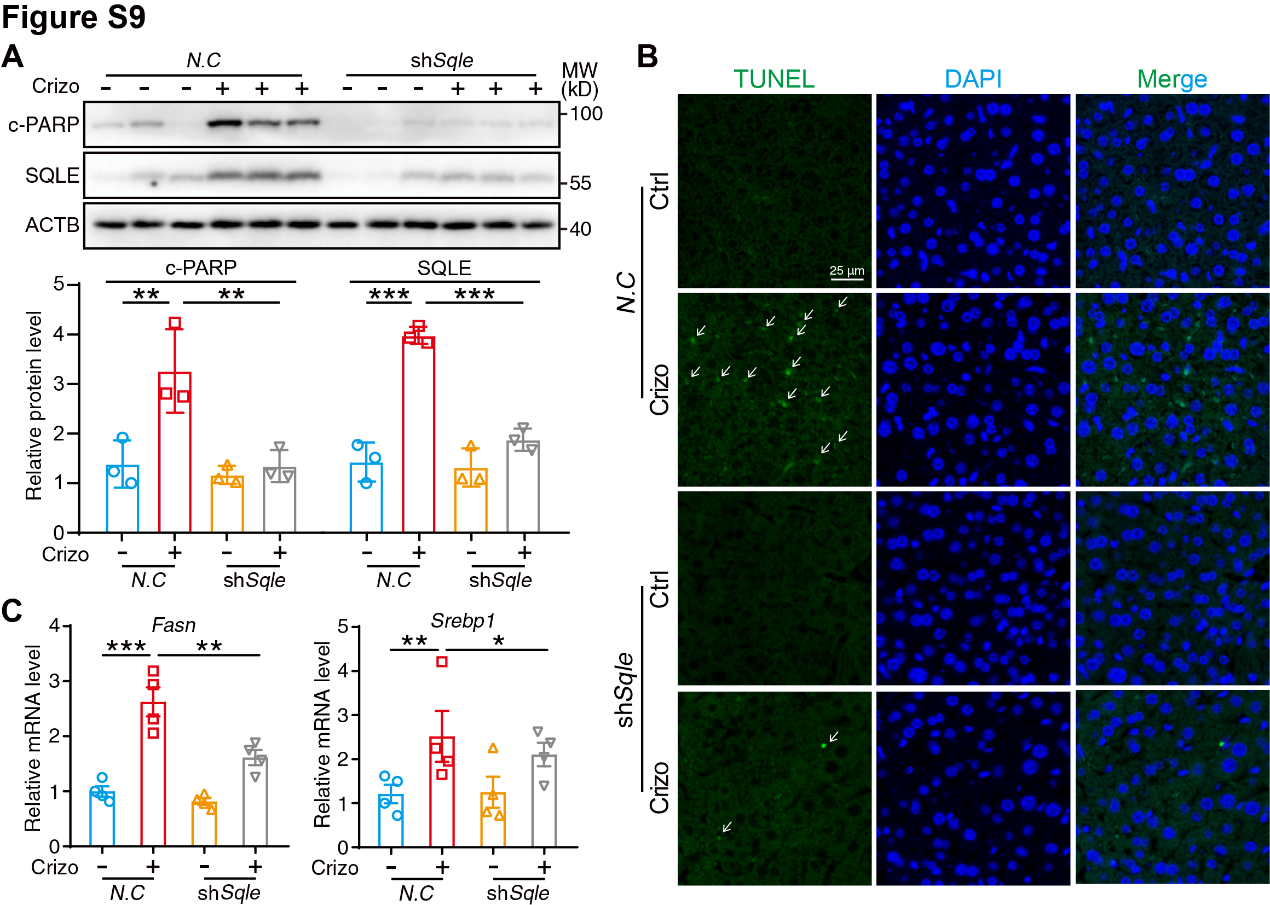


**Figure S9. *Sqle* knockdown by AA8-TBG promoter-sh*Sqle* could ameliorate with crizotinib-induced hepatotoxicity. (A-C)** The livers from four groups, including *N.C* + Ctrl, *N.C* + Crizotinib, sh*Sqle* + Ctrl, and sh*Sqle* + Crizotinib, were harvested. **(A)** Relative expressions of c-PARP and SQLE were analyzed by western blot with ACTB as a loading control (n = 3 per group). **(B)** The mRNA levels of *Fasn* and *Srebp1* were analyzed by qPCR with ACTB as an internal reference (n = 4 per group). **(C)** Representative fluorescence microscope images of liver tissues stained with TUNEL and DAPI were presented. Scale bar = 25 µm. White arrows indicated apoptotic cells. The results are presented as the mean ± SD. The *P* value was calculated by one-way ANOVA (Dunnett’s multiple comparisons test). **P* < 0.05; ***P* < 0.01; ****P* < 0.001.


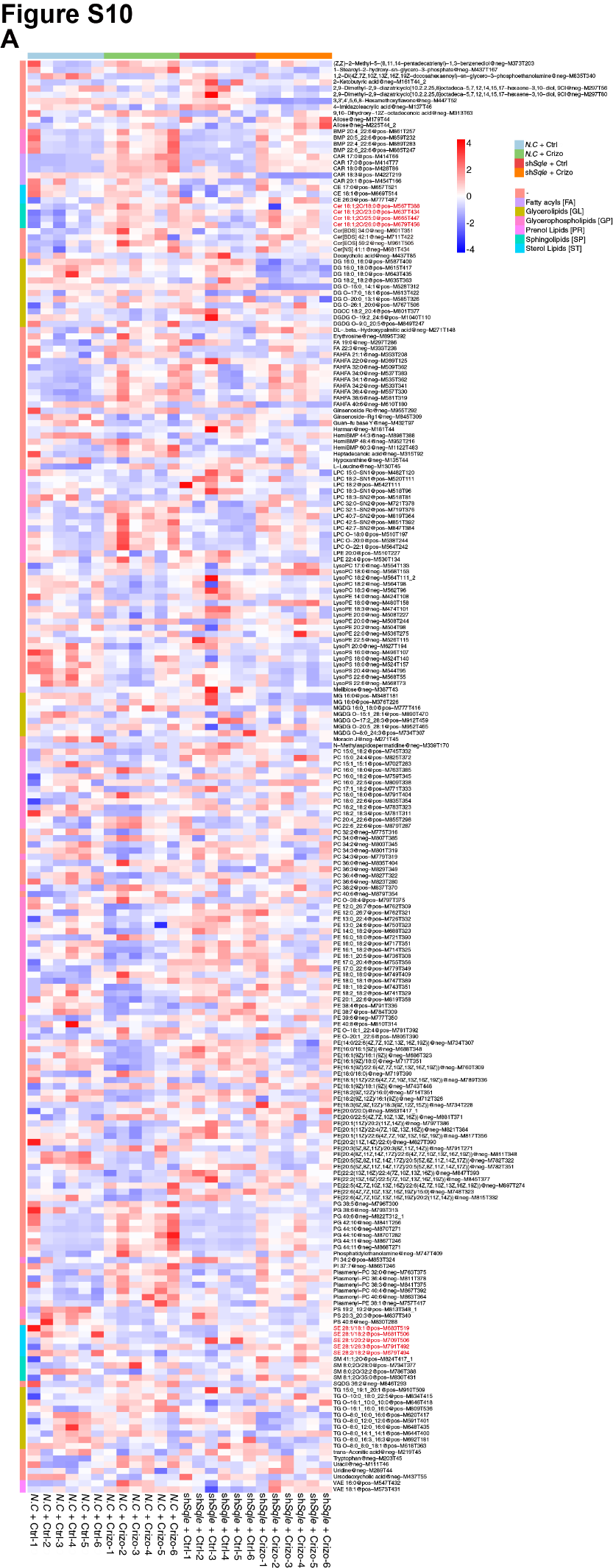


**Figure S10. SQLE is associated with extensive metabolites changes. (A)** Untargeted metabolomic analysis was used to determine the alteration of hepatic metabolites within four groups, including *N.C* + Ctrl, *N.C* + Crizotinib, sh*Sqle* + Ctrl, and sh*Sqle* + Crizotinib (n = 6 per group). Clustering heatmap analysis of metabolites expression within four groups, colors depicted the intensity of response. The red font represented sphingolipid-related metabolites.


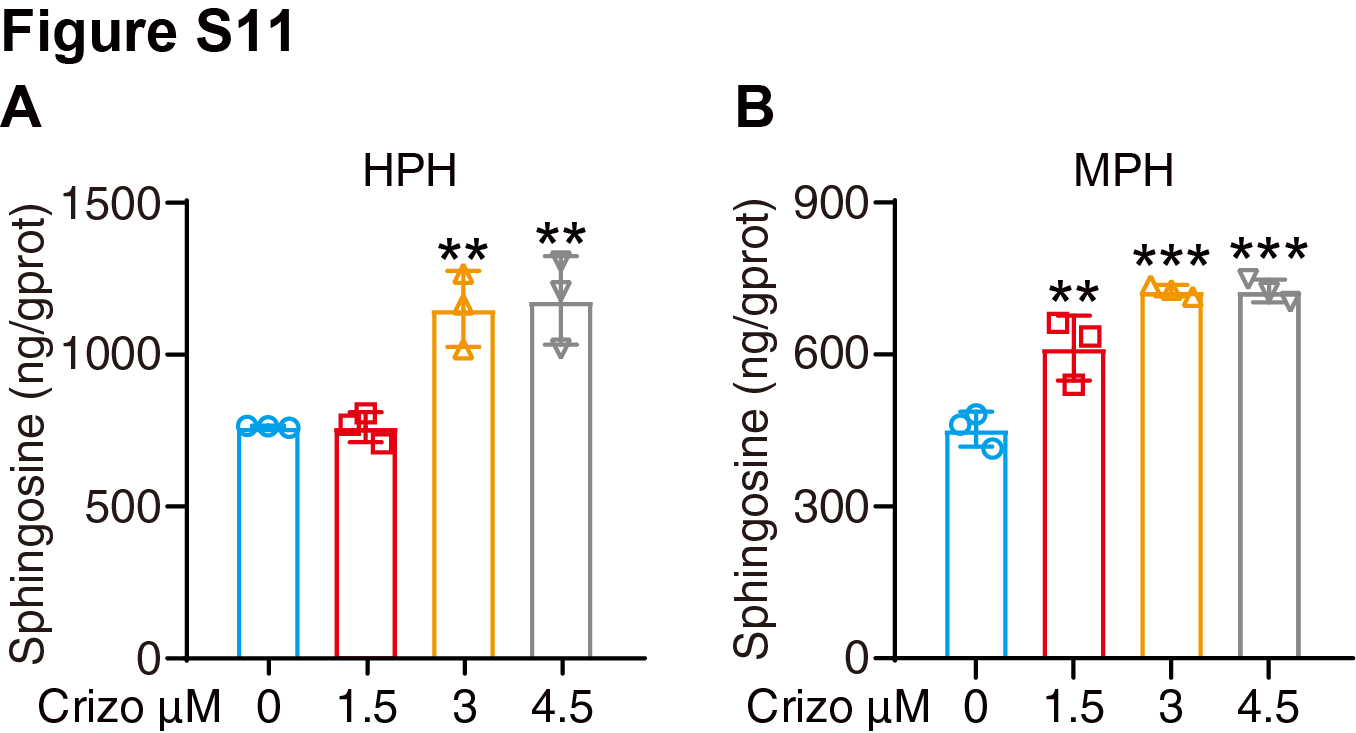


**Figure S11. Crizotinib increases intracellular sphingosine levels in human primary hepatocytes and mouse primary hepatocytes. (A)** Human primary hepatocytes (HPH) from 3 donors were treated with 0, 1.5, 3 and 4.5 μM crizotinib for 24 h. The intracellular levels of sphingosine were analyzed by ELISA assay. (**B**) Mouse primary hepatocytes (MPH) were treated with 0, 1.5, 3 and 4.5 μM crizotinib for 24 h. The intracellular levels of sphingosine were analyzed by ELISA assay (n = 3 independent replicates). The results are presented as the mean ± SD. The *P* value was calculated by one-way ANOVA (Dunnett’s multiple comparisons test). ***P* < 0.01; ****P* < 0.001.


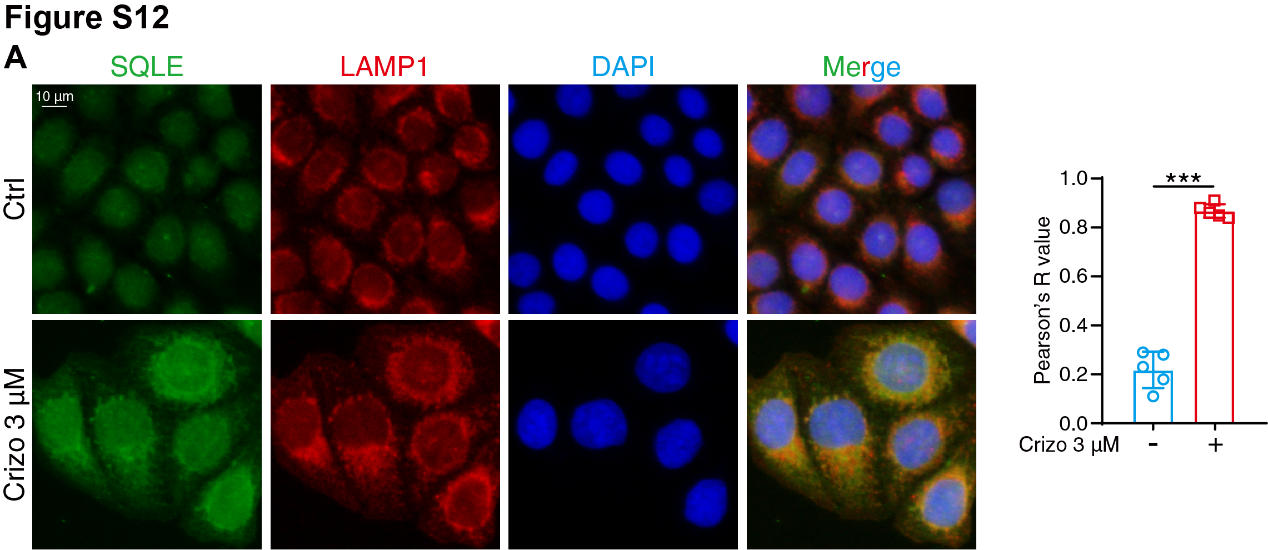


**Figure S12. Crizotinib enhances the colocalization between SQLE and LAMP1 in HL-7702 cells. (A)** HL-7702 cells were treated with or without 3 μM crizotinib. Representative fluorescent images of cells stained with SQLE (green), LAMP1 (red) and DAPI (blue) were presented. The Pearson’s R value was analyzed with Fiji software (n = 5 fields). The results are presented as the mean ± SD. The *P* value was calculated by Student’s *t* test (unpaired, two-tailed, 2 groups). ****P* < 0.001.


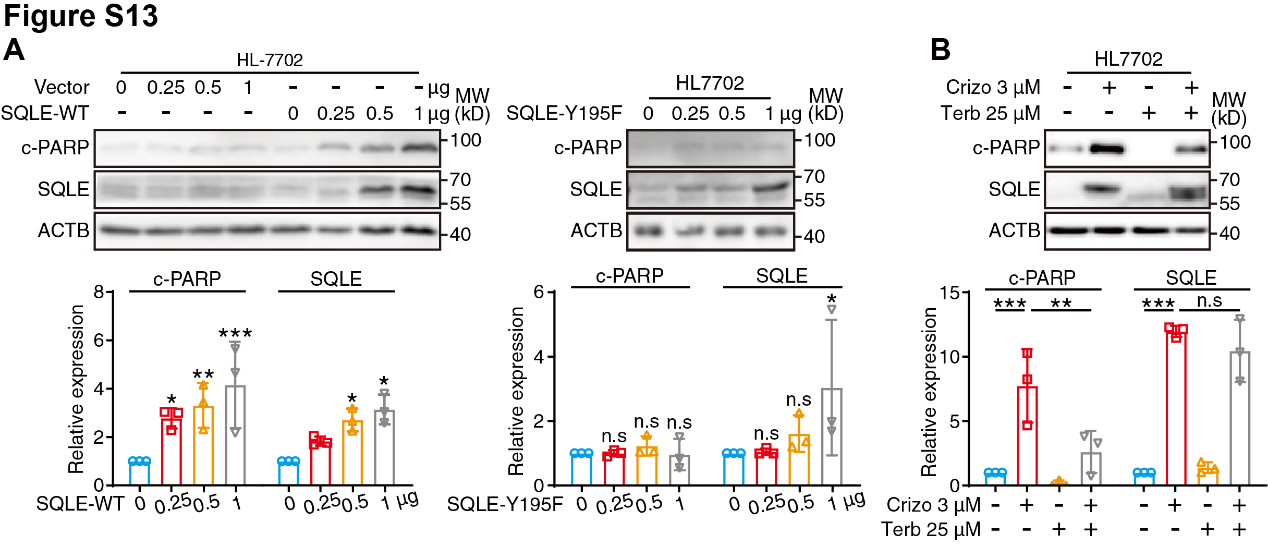


**Figure S13. SQLE is positively related with apoptosis which depended on its enzymatic function. (A)** HL-7702 cells were transfected with vector, wild-type SQLE or mutant-Y195F-SQLE plasmids in an amount-dependent manner for 24 h. Relative expressions of c-PARP and SQLE were analyzed by western blot with ACTB as a loading control (n = 3 independent replicates). **(B)** HL-7702 cells were treated with 3 μM crizotinib with or without 25 μM terbinafine for 24 h. Relative expressions of c-PARP and SQLE were analyzed by western blot with ACTB as a loading control (n = 3 independent replicates). The results are presented as the mean ± SD. The *P* value was calculated by one-way ANOVA (Dunnett’s multiple comparisons test). n.s = no significance; **P* < 0.05; ***P* < 0.01; ****P* < 0.001.


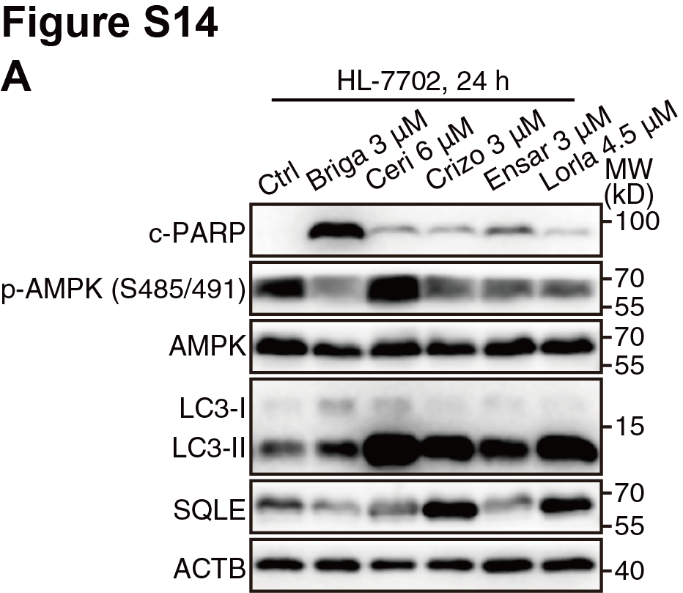


**Figure S14. Comparative toxicity mechanism analysis with known ALK inhibitors. (A)** HL-7702 cells were treated with 3 μM brigatinib, 6 μM ceritinib, 3 μM crizotinib, 3 μM ensartinib, and 4.5 μM lorlatinib for 24 h (The research concentrations were 3 times clinically Cmax for each ALK inhibitor). Relative expressions of c-PARP, p-AMPK (S485/491), AMPK, LC3, and SQLE were analyzed by western blot with ACTB as a loading control.

**Supplementary table**

**Supplementary table 1. Characteristics of the human primary hepatocytes**

| **Donor** | **HPH-1** | **HPH-2** | **HPH-3** |
| --- | --- | --- | --- |
| Product | M00995-P | M00995-P | F00995-P |
| Lot | HVN**** | QBU**** | XSM**** |
| Gender  Age  BMI | Male | Male | Female |
|  | 33 | 55 | 59 |
|  | 31.0 | 25.9 | 27.1 |
| EBV  RPR  CMV | IgG+ | IgG+ | IgG+ |
|  | Neg | Neg | Neg |
|  | Neg | Pos | Pos |
| COD  General  Meds | Anoxia 2^nd^ to Natural Causes/Cardiovascular | ICH | CVA |
|  | Depression, right wrist and ligament repair s/p MVC 7 yrs ago. | Hypertension × 5 yr, Diabetes × 1 yr, Skin Cancer × unknown duration | Urethra surgery (short urethra w/ frequent UTIs). |
|  | Celexa, Seroquel | Celexa, Seroquel | None |
| Alcohol  Tobacco  Drug | Beer:1-2 times/week, 6 pack on weekends, 3-4 on weekdays for 12 years | Socially | None reported |
|  | 0.5-1 ppd cigarettes × 15 yrs, current | None | 1 ppd x 40 yrs |
|  | Meth for 6 mos, used 1-2 times/week, last used 7 years ago, IVDA 7 yrs ago | None | None reported |

**HPH represents human primary hepatocytes**
